# Gammaherpesvirus infection triggers the formation of tRNA fragments from premature tRNAs

**DOI:** 10.1128/mbio.00875-25

**Published:** 2025-05-30

**Authors:** Aidan C. Manning, Mahmoud M. Bashir, Kyle Rapchak, Calyssa J. Huff, Ariana R. Jimenez, Sheila Gonzalez, Courtney L. Woodruff, Heather E. Upton, Kathleen Collins, Todd M. Lowe, Jessica M. Tucker

**Affiliations:** 1 Department of Biomolecular Engineering, Baskin School of Engineering, University of California Santa Cruz 189230, Santa Cruz, California, USA; 2 Department of Microbiology and Immunology, Carver College of Medicine, University of Iowa 311821 , Iowa City, Iowa, USA; 3 Department of Molecular and Cell Biology, University of California Berkeley 196203, Berkeley, California, USA; Washington University in St. Louis, St. Louis, Missouri, USA; University of Pennsylvania Perelman School of Medicine, Philadelphia, Pennsylvania, USA

**Keywords:** virology, herpesviruses, tRNA, tRNA fragments, tRNA splicing, viral tRNA

## Abstract

Transfer RNAs (tRNAs) are fundamental for both cellular and viral gene expression during viral infection. In addition, mounting evidence supports the biological function for tRNA cleavage products, including the control of gene expression during conditions of stress and infection. We previously reported that infection with the model murine gammaherpesvirus 68, MHV68, leads to enhanced tRNA transcription. However, whether this has any influence on tRNA transcript processing, viral replication, or the host response is not known. Here, we combined two new approaches, sequencing library preparation by ordered two template relay (OTTR) and tRNA bioinformatic analysis by tRAX, to quantitatively profile full-length tRNAs and tRNA fragment (tRF) identities during MHV68 infection. We find that MHV68 infection triggers both pre-tRNA and mature tRNA cleavage, resulting in the accumulation of specific tRFs. OTTR-tRAX revealed not only host tRNAome changes, but also the expression patterns of virally encoded tRNAs (virtRNAs) and virtRFs made from the MHV68 genome. Because the transcript ends of several host tRFs matched tRNA splice junctions, we tested and confirmed the role of tRNA splicing factors TSEN2 and CLP1 in MHV68-induced tRF biogenesis. Further, we show that CLP1 kinase, which regulates tRNA splicing among other RNA processing events, is required for efficient MHV68 replication. Our findings provide new insight into how gammaherpesvirus infection both impacts and relies on tRNA transcription and processing.

**IMPORTANCE:**

Diverse conditions of infection and cellular stress incite the cleavage of transfer RNAs (tRNAs), leading to the formation of tRNA fragments (tRFs) that can directly regulate gene expression. In our study of gammaherpesviruses, such as murine herpesvirus 68 and human oncogenic Kaposi sarcoma-associated herpesvirus, we discovered that tRNA regulation and cleavage are key components of gene reprogramming during infection. We present the first in-depth profile of tRF generation in response to DNA virus infection, using state-of-the-art sequencing techniques that overcome several challenges with tRNA sequencing. We present several lines of evidence that tRFs are made from newly transcribed premature tRNAs and propose that this may be a defining characteristic of tRNA cleavage during infection. Finally, we show that tRNA splicing machinery is involved with the formation of some MHV68-induced tRFs, with a key regulator of splicing, CLP1, required for maximal viral titer. Taken together, we posit that tRNA processing may be integral to the elegant shift in gene expression that occurs during viral takeover of the host cell.

## INTRODUCTION

Transfer RNAs (tRNAs) are important non-coding RNAs with dual functionality, essential for protein translation and, when processed, as rich sources of functional small RNAs called tRNA fragments (tRFs). Advances in small RNA sequencing technology have revealed an abundance of tRFs made by endonucleolytic cleavage of tRNAs, which typically increase in abundance in response to cellular stress, viral infection, and other disease states (1–6). While some cleavage products may solely be degradation intermediates, some tRFs have been shown to have protein binding partners that would impart functionality, such as Argonaute or YBX1 (7, 8). Diverse classes of tRFs can be made from tRNAs—fragments of differing lengths can be generated from the 5′ end, 3′ end, or internal region of the tRNA, with sequence diversity arising from the parental tRNA family origin. The diversity in sequence and structure has resulted in overlapping naming systems for tRFs, including tRNA halves, tRNA-derived RNAs (tDRs) (9), and tiRNAs (10), among others. Notably, tRF diversity explains the numerous functions and protein interactors observed for tRFs (7, 8, 11). Comprehensively identifying tRFs produced in response to stress and infection is fundamental to understanding the role tRFs play in different cellular responses.

Viral infection involves an overhaul of host gene expression and thus represents a system in which tRNA regulation likely plays a significant role. We have previously explored the tRNA expression landscape during infection using murine gammaherpesvirus 68 (MHV68), which is a large double-stranded DNA virus genetically similar to two cancer-causing human viruses, Kaposi sarcoma-associated herpesvirus (KSHV) and Epstein-Barr virus (EBV) (4). Due to the strict human tropism of KSHV and EBV, MHV68 has served as an essential model for understanding gammaherpesvirus pathogenesis and host antiviral control (12). MHV68 uniquely encodes eight viral tRNA-miRNA encoded RNAs (TMERs) that are important for pathogenesis (13–15). Each TMER encodes a viral tRNA (virtRNA) followed by one or two pre-miRNA stem-loops (SLs).

Expression of the viral miRNAs requires host tRNA transcription and processing machinery, including RNA polymerase III and tRNAseZ/ELAC2 (13, 14, 16). Because of its genetic similarity to human gammaherpesviruses and its exploitation of tRNA expression machinery, we reasoned that MHV68 infection serves as an ideal model for elucidating novel mechanisms of tRNA transcription and processing in mammalian cells. Our previous tRNA profiling of MHV68-infected fibroblasts demonstrated selective upregulation of approximately 20% of host tRNA genes; however, no patterns regarding specific tRNA families emerged (4). Additionally, differential expression of tRNAs during infection manifested primarily at the level of premature tRNA (pre-tRNA) transcripts with minor changes in mature tRNA pools (<2-fold). This work implied that the efficiency of tRNA processing and maturation is decreased during MHV68 infection, but technical limitations of the sequencing method employed (DM-tRNA-seq) (17) hindered our ability to explore shorter derivatives of tRNAs in the sample.

Mounting evidence suggests that tRFs can functionally impact the replication of diverse RNA viruses, including retroviruses, respiratory syncytial virus, and hepatitis C virus, through binding and regulating viral and host RNAs and proteins (2, 5, 18). Inspired by these emerging indications of tRF roles in virally infected cells, we profiled host and viral tRFs produced in response to DNA virus infection. We applied recently described library preparation and tRNA mapping methodologies, in a strategy we refer to here as OTTR-tRAX, to small RNA isolated from MHV68-infected murine fibroblasts. OTTR-tRAX recapitulated the pre-tRNA upregulation in response to MHV68 infection (4). It also revealed widespread tRF production from both host and viral tRNAs (virtRNAs) as a molecular signature of gammaherpesvirus infection.

Surprisingly, we found that many tRFs are derived from pre-tRNAs. Some pre-tRFs match expected tRNA splicing intermediates. Indeed, we link their presence to the tRNA splicing endonuclease, TSEN2, as well as the tRNA splicing regulatory RNA kinase, CLP1. We show that TSEN2 and CLP1 promote the generation of 5′ pre-tRFs derived from pre-tRNA-Tyr.

Further, we show that CLP1 is required for efficient MHV68 replication. Altogether, we provide evidence that tRNA regulation by processing impacts DNA virus infectivity.

## MATERIALS AND METHODS

### Cell lines and culturing conditions

NIH 3T3 murine fibroblasts (ATCC CRL-1658), NIH 3T12 murine fibroblasts (ATCC CCL-164), and MC57G fibrosarcoma cells (ATCC CRL-2295) were maintained in high-glucose Dulbecco’s modified Eagle’s medium (DMEM; Gibco 11965092) supplemented with 10% fetal bovine serum (FBS; Gibco 26140079). Cultures were screened regularly for mycoplasma (Invivogen) and cultured in the absence of antibiotics. MC57G, which are C57BL/6-derived and more closely match the mouse genome GRCm38/mm10, were used for sequencing. NIH 3T3s (NIH/Swiss-derived) have a higher transfection efficiency than MC57Gs and thus were used for experiments involving genetic manipulation. Both fibroblast cell lines respond similarly to MHV68 infection with regard to tRNA regulation, as described in Results. NIH 3T12s are used for virus propagation. The lentiviral vector used to overexpress *Clp1* and *Tsen2* in our study, pLV[Exp]-mCherry:T2A:Puro-EF1A>mTsen2 and pLV[Exp]-mCherry:T2A:Puro-EF1A>mClp1 were constructed by VectorBuilder. The vector IDs are VB240824-1026afx and VB240824-1025aas, which can be used to retrieve detailed information about the vector on vectorbuilder.com. An mCherry-expressing lentiviral vector was used as an empty vector control. Lentiviruses were packaged with third-generation packaging plasmids in HEK293Ts and used to transduce NIH 3T3s. Overexpression lines were selected with 2 µg/mL puromycin.

### Virus infections

Green fluorescent protein (GFP) expressing MHV68-R443I carrying a single point mutation in the viral endonuclease muSOX and the MHV68-MR mutant revertant were amplified in NIH 3T12s (19, 20, 20). The MHV68-MR was used as the source of wild-type MHV68 for all experiments in this work. MHV68-infected NIH 3T12 cells and media were subjected to two freeze-thaws, then cellular material was pelleted and discarded. Viruses present in the supernatant were pelleted at high speed (12,000 × *g*) for 2 h, then treated with DNase (Fisher #EN0523) for 1 h at 37°C. Media were added, and virus was pelleted at high speed (12,000 × *g*) for 2 h. Virus pellet was dissolved in DMEM + 10% FBS media, aliquoted, and stored at −80°C. MHV68-GFP stock titer was measured on NIH 3T3 cells for the 50% tissue culture infective dose (TCID_50_). For infections, MHV68 virus was added to ½ culture volume of media, placed on NIH 3T3 or MC57G cells as indicated for 1.5 h at multiplicity of infection (MOI) of 0.05 or 5 (as calculated by TCID_50_) to allow viral entry, then replaced with fresh DMEM + 10% FBS medium.

### Cell death assays

Zombie Violet (BioLegend) was used to stain dead cells at a 1:300 dilution in PBS for 30 min. The cells were washed two times in FACS buffer (1% FBS in PBS) and analyzed on a CytoFLEX (Beckman Coulter). LDH assays were performed with an LDH-Glo Cytotoxicity Assay (Promega) with a 1:200 dilution of growth media and according to the manufacturer’s instructions.

### siRNA treatment

Pooled siRNA (sequences in Table 1) purchased from Dharmacon. For knockdown experiments in Fig. 7, we nucleofected using a Neon Transfection System (Thermo Fisher MPK5000), using 200 nM siRNAs (as recommended by the manufacturer) at 1,400 volts/20 ms/2 pulses into 5 × 10^5^ NIH 3T3 cells/well and plated in a six-well dish. For experiments in Fig. 8, 1.5 × 10^5^ cells/well were seeded 24 h prior into a six-well dish and then transfected with 60 nM (final concentration) siRNAs using RNAiMAX. The cells were infected 24 h post-nucleofection or transfection and harvested 24 h post-infection (hpi). Total RNA was analyzed via reverse transcription-quantitative polymerase chain reaction (RT-qPCR).

**TABLE 1:** Oligonucleotide sequences

Primer	Sequence(s)
RNA oligos for SL-qPCR validation	
Mm pre-tRNA-Tyr	5′-rUrArUrCrUrCrCrCrUrUrCrGrArUrArGrCrUrCrArGrUrUrGrGrUrArGrArGrCrGrGrArGrGrArCrU rGrUrArGrUrArUrArGrGrUrGrUrUrGrArUrArUrCrCrUrUrArGrGrUrCrGrCrUrGrGrUrUrCrGrArArU
Mm 5′ pre-tRF-Tyr	5′-rUrArUrCrUrCrCrCrUrUrCrGrArUrArGrCrUrCrArGrUrUrGrGrUrArGrArGrCrGrGrArGrGrArCrU rGrUrArG
Pooled siRNA sequences (Dharmacon)	
Non-targeting siRNA pool; Horizon D-001810–10-05	UGGUUUACAUGUCGACUAA, UGGUUUACAUGUUGUGUGA, UGGUUUACAUGUUUUCUGA, UGGUUUACAUGUUUUCCUA
Mouse Clp1 siRNA pool; Horizon L-051374-02-0005	CUACUUACGUGGAGUUAGA, GAAGCCAACGACUAAGUUU, AGACAACCAGCUCAAGUUA, AGACAACCAGCUCAAGUUA
Mouse Tsen2 siRNA pool; Horizon L-050573-01-0005	CUAUGAGAGUUACGAGUCA, CAUGCAAGCUACUCGGUCA, UGAGCAGAGUCUCGGGGAA, GGAAGGGUUACUUUGGAAA
Northern probes	
Mm-tRNA-Tyr-GTA-1-3_probe	5′-CTACAGTCCTCCGCTCTACCA
5S_probe	5′-AGCCTACAGCACCCGGTATT
Primers used for reverse transcription	
5′tRF-Arg-TCT-3-1_RT	5′-CTCAACTGGTGTCGTGGAGTCGGCAATTCAGTTGAGTAGAAGTC
5′tRF-Gln-CTG_RT	5′-CTCAACTGGTGTCGTGGAGTCGGCAATTCAGTTGAGTGCTAACC
5’tRF-Leu-CAG-3_RT	5′-CTCAACTGGTGTCGTGGAGTCGGCAATTCAGTTGAGTCCTTAGA
5′tRF-Tyr-GTA-1-3_RT	5′-CTCAACTGGTGTCGTGGAGTCGGCAATTCAGTTGAGCTACAGTC
Primers used for qPCR	
Mm-tRNA-Tyr-GTA-1-3_qPCRf	5′-CCTTCGATAGCTCAGTTGGTAGAGC
Mm-tRNA-Tyr-GTA-1-3_qPCRr	5′-GGATTCGAACCAGCGACCTAAGGATATC
5′tRF-Arg_ TCT-3-1_qPCRf	5′-GGCTCTGTGGCGCAATGG
5′tRF-Gln-CTG_qPCRf	5′-GGTTCCATGGTGTAATGGT
5′tRF-Leu-CAG-3_ qPCRf	5′-GTCAGGATGGCCGAGTGG
5′tRF-Tyr-GTA-1–3_ qPCRf	5′-CCTTCGATAGCTCAGTTGGTAGAGC
SL-univ_qPCRr	5′-GTGTCGTGGAGTCGGC
Mm_Clp1_qPCRf	5′-ATGAGCGAGGAATCCAATGATG
Mm_Clp1_qPCRr	5′-CTCCAACTGAACCGATTGAGAG
Mm_Tsen2_qPCRf	5′-CCAAACTTCACAATCGCCAAC
Mm_Tsen2_qPCRr	5′-AGTGGCTCTGTGTAGTCTGTAA
MHV68_Orf50_qPCRf	5′-GGCCGCAGACATTTAATGAC
MHV68_Orf50_qPCRr	5′-GCCTCAACTTCTCTGGATATGCC
MHV68_gB_qPCRf	5′-GGCCCAAATTCAATTTGCCT
MHV68_gB_qPCRr	5′-CCCTGGACAACTCCTCAAGC
18S_qPCRf	5′-GTGGAGCGATTTGTCTGGTT
18S_qPCRr	5′-CGCTGAGCCAGTCAGTGTAG
U6_qPCRf	5′-TCGCTTCGGCAGCACATATAC
U6_qPCRr	5′-AATATGGAACGCTTCACG

### RT-qPCR and SL-qPCR

Total RNA was isolated from cells using TRIzol (Invitrogen) and was treated with Turbo DNase (Invitrogen AM2239). Turbo-treated RNA was then reverse transcribed with AMV RT (Promega M5108) or SuperScript-III (Thermo Fisher 18080044) primed with random 9-mer (IDT) for total RNA measurements by RT-qPCR or with AMV RT primed with SL primers for 5′ tRF measurements by SL-qPCR. Binding of SL primers requires an exact 3′ end for successful amplification; thus, we designed primers using the most frequent 3′ end of upregulated 5′ tRFs in our data set. Our SL-qPCR assays will detect the following using tDRnamer (9) nomenclature using mm10 as a sequence source: tDR-1:37-Tyr-GTA-1-M2, tDR-1:37-Arg-TCT-3-1, and tDR-1:24-Gln-CTG-1-M3. Gene expression was measured by amplifying cDNA using iTaq SYBR Green Supermix (Bio-Rad 1725125) on a QuantStudio 3 (Applied Biosystems), and all primers are listed in Table 1. RT-qPCR was analyzed using the delta delta CT (ΔΔCT) method. Average fold changes were calculated relative to 18S or U6 internal controls.

### Northern analysis

Total RNA was loaded onto 8–12% PAGE-7 M urea gels and transferred to Hybond-N+ (GE) membranes using a Trans-Blot Turbo Transfer System (Bio-Rad) in 1× TBE. Blots were crosslinked using the auto-crosslink setting on a UV Crosslinker FB-UV-XL-1000 (1,200 μJ × 100) (Fisher Scientific) and prehybridized in ULTRAhyb buffer (Thermo Fisher) at 42°C for 1 h before adding radiolabeled probe. Probes were generated by end-labeling oligonucleotides specific for Tyr or 5S (Table 1) as a loading control using T4 PNK and [γ-32P]ATP. Blots were probed overnight at 55°C and washed three times for 10 min in 0.5× SSC (1× SSC is 0.15 M NaCl plus 0.015 M sodium citrate) and 0.1% SDS. Blots were imaged using a Typhoon imager and processed on Photoshop (Adobe). Densitometry was performed using Bio-Rad ImageLab software.

### Ordered two template relay sequencing (OTTR-seq)

RNAs were isolated from mock-infected, MHV68-MR-infected, or MHV68-R443I-infected MC57G (MOI = 5, 24 h) in biological triplicate using miRVANA isolation kits (Thermo Fisher) and were used for generating OTTR-seq libraries (21). Briefly, deacylated, CIP-treated, and PNK-treated small RNA was 3′ tailed using mutant BoMoC RT in buffer containing only ddATP for 90 min at 30°C, with the addition of ddGTP for another 30 min at 30°C. This was then heat-inactivated at 65°C for 5 min, and unincorporated ddATP and ddGTP were hydrolyzed by incubation in 5 mM MgCl_2_ and 0.5 units of shrimp alkaline phosphatase (rSAP) at 37°C for 15 min. 5 mM EGTA was added and incubated at 65°C for 5 min to stop this reaction. Reverse transcription was then performed at 37°C for 30 min, followed by heat inactivation at 70°C for 5 min. The remaining RNA and RNA/DNA hybrids were then degraded using 1 unit of RNase A at 50°C for 10 min. cDNA was then cleaned up using a MinElute Reaction CleanUp Kit (Qiagen). To reduce adaptor dimers, cDNA was run on a 9% urea PAGE gel, and the size range of interest was cut out and eluted into gel extraction buffer (300 mM NaCl, 10 mM Tris; pH 8.0, 1 mM EDTA, 0.25% SDS) and concentrated using EtOH precipitation. Size-selected cDNA was then PCR amplified for 12 cycles using Q5 High-Fidelity Polymerase (NEB #M0491S). Amplified libraries were then run on a 6% TBE gel, and the size range of interest was extracted to reduce adaptor dimers further. Gel slices were eluted into gel extraction buffer (300 mM NaCl, 10 mM Tris; pH 8.0, 1 mM EDTA) followed by concentration using EtOH precipitation. Final libraries were pooled and sequenced using 150 SE and 200 cycle kit on an Illumina NovaSeq.

### tRNA Analysis of eXpression (tRAX)

Sequencing adaptors were trimmed from raw reads using cutadapt, v1.18, and read counts were generated for host and viral small RNA types using tRAX (22). Briefly, trimmed reads were mapped to a combined mouse (GRCm38/mm10) and gammaherpesvirus (MHV68) reference containing mature tRNAs obtained from GtRNAdb (23) and their corresponding genomic sequences (for mapping sequences derived from pre-tRNAs) using Bowtie2 in very-sensitive mode with the following parameters to allow for a maximum of 100 alignments per read: --very- sensitive --ignore-quals --np 5k 100. Mapped reads were filtered to retain only the “best mapping” alignments. Raw read counts of tRNAs and other small RNA types were computed using tRNA annotations from GtRNAdb, and annotations from GENCODE M23 and miRBase v22 (24). A full description of the mapping parameters for tRFs can be found here (22). In brief, reads are first mapped to a custom-built reference database for mature tRNAs, including the fully processed tRNA sequences with 3′ CCA tails. For mature tRNA reads, if the read covers both the 5′ and 3′ boundaries of the mature tRNA, it is considered a full-length mature tRNA. For tRFs, tRAX computes separate read counts for tRFs that are within 10 nt of the 5′ end, 3′ end, or internal sequences that are not within 10 nt of either end (other) of mature tRNA sequences. In contrast, if sequencing reads have at least 5 nt that extend beyond the 5′ and 3′ boundaries of the mature tRNA (only one extension is required), they map best to the genomic database and are binned as pre-tRNAs. Within pre-tRNA reads, if both transcript ends extend at least 2 nt beyond the 5′ and 3′ boundaries, the read is assigned as a full-length pre-tRNA. Otherwise, it is assigned as a partial pre-tRNA. Partial pre-tRNAs are not further binned, so the 5′ pre-tRFs discussed in this manuscript were manually extracted and identified using tDRnamer (9). We note that it is not possible to distinguish whether tRFs binned as “5′,” “3′,” or “other” came from a premature or mature tRNA, unless an intron or 3′ CCA sequences are present. Raw read counts were then normalized and compared using DESeq2 and its associated statistical tests.

### Statistical analysis

All experiments were performed in at least biological triplicate, meaning that experiments were performed on different days with different cell populations in terms of stock vials or passage numbers. All RT-qPCR statistical analysis was performed using raw ΔCt values. The statistical test used is indicated in the figure legends.

## RESULTS

### Quantitative profiling of tRNAs and tRFs during MHV68 infection

To quantitatively profile tRNA and tRF expression during MHV68 infection, we considered new methodologies to enhance the accuracy and throughput of combined tRNA/tRF sequencing, which is inherently difficult due to both technical and bioinformatic challenges.

**Fig 1:**
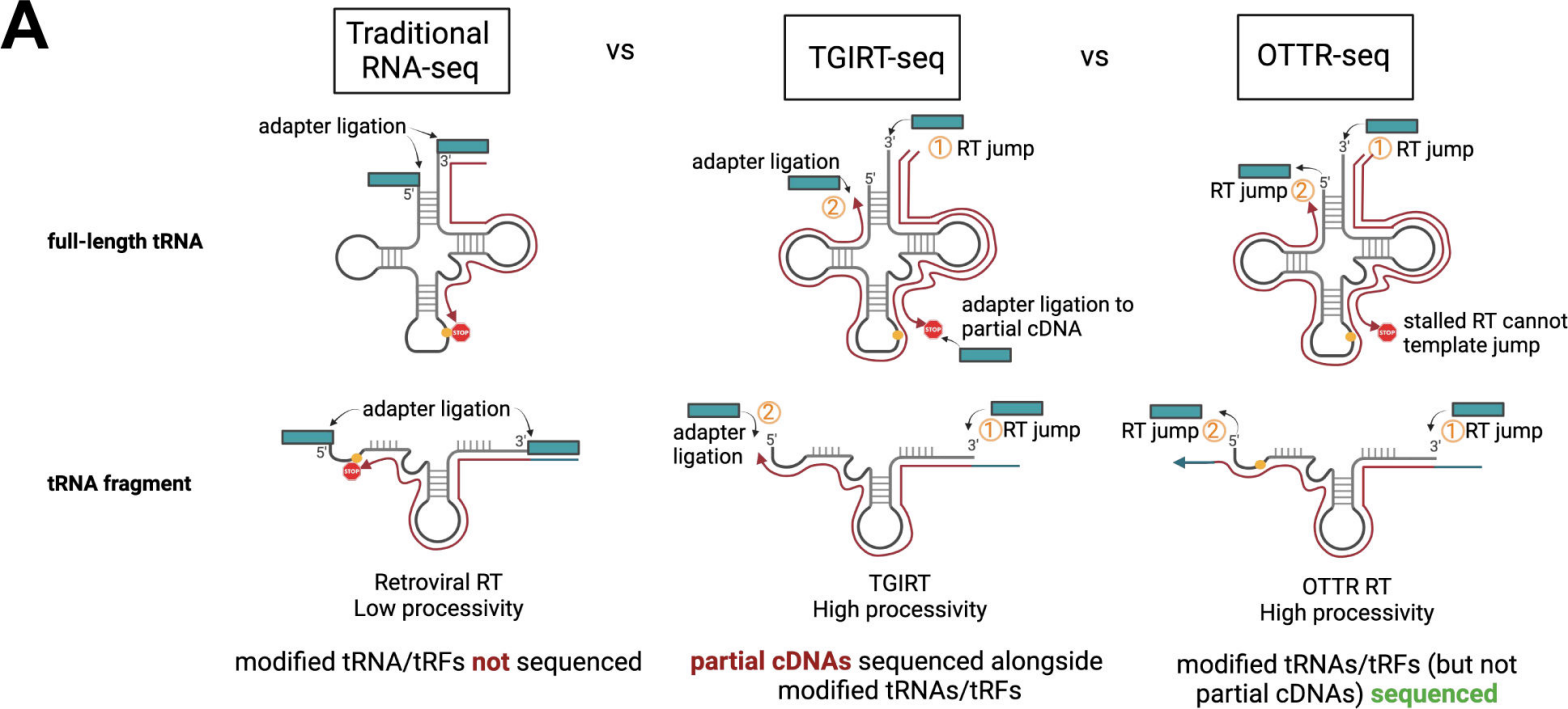
Benefits of OTTR-seq for tRNA sequencing. Traditional RNA-seq uses retroviral reverse transcriptases (RTs) with low processivity through tRNA modifications, resulting in depletion of modified transcripts in final sequencing libraries. TGIRT-seq uses the more processive TGIRT enzyme but allows partial/incomplete cDNA products into library assembly where they are indistinguishable from biologically relevant tRFs. The high processivity and template jumping activity of the OTTR RT used in OTTR-seq ensures that highly modified tRNAs and genuine tRFs (and not partial cDNA products) are sequenced.

Standard small RNA library preparations use retroviral reverse transcriptases (RTs) that lack sufficient processivity to reverse transcribe highly modified and structured tRNAs and tRFs from end to end (Fig. 1) (25). Coverage of full-length tRNAs is dramatically improved with the use of more processive RTs, such as TGIRT (26) used in DM-tRNA-seq (17). TGIRT uses a template-jumping mechanism to initiate reverse transcription, adding the first sequencing adaptor during cDNA production. This eliminates the need to ligate adaptors prior to reverse transcription, which is convenient for low RNA input and workflow. However, this workflow allows partial cDNA resulting from disengagement of the RT at modified bases into the sequencing pool; thus, incomplete cDNA products are indistinguishable from biologically relevant tRFs.

Because of these technical issues, it is currently standard practice to analyze full-length tRNAs and tRFs using separate library preparation techniques, such as DM-tRNA-seq for full-length tRNAs and ARM-seq for tRFs (17, 27). To overcome these challenges and facilitate a single approach to monitor both tRNAs and tRFs by sequencing, we applied OTTR (21) to prepare tRNA sequencing libraries. OTTR harnesses the template jumping activity and high processivity of an engineered eukaryotic retrotransposon RT, called BoMoC, to add both adapters to RNA sequentially by template-jumping (21, 28). Processivity through the length of the tRNA or tRF is required for the 2nd adaptor addition (Fig. 1). Thus, this technology is well-suited for simultaneous tRNA and tRF analysis, as it should minimize and exclude partial cDNA synthesis products from the sequenced pool. In comparison, TGIRT template-jumping activity is used for the inclusion of the initial 5′ adaptor, but the 3′ adaptor is added via ligation, thus allowing incomplete cDNA products into the sequencing library. We have combined this technology with best-practice and user-friendly tRNA and tRF mapping software, called tRAX (22), into a pipeline we refer to here as “OTTR-tRAX.”

We performed OTTR-tRAX on small RNA extracted in biological triplicate from the murine fibroblast cell line MC57G with or without a single round of infection with MHV68 (24 h) at an MOI = 5, conditions we have previously analyzed by DM-tRNA-seq (4). We note that our choice to perform RNA sequencing from MC57G was based on pilot studies in which we observed better mapping efficiency to GRCm38/mm10 using MC57G, which is C57BL/6 derived, compared to NIH 3T3, which is NIH/Swiss derived. We performed steps to deacylate (treatment at pH = 9), remove 5′-P (CIP treatment), and remove 3′ cyclic phosphates (PNK treatment) from tRNAs and tRFs to offset potential interference of these modifications during cDNA library preparation. To account for the known bias of PCR to amplify shorter fragments, the final cDNA library was size selected, PCR amplified, and sequenced in separate pools corresponding to 15–50 or 50–200 nt inserts. We analyzed our sequencing data using the tRAX software package for the analysis of tRNAs and tRFs (see Materials and Methods).

**Fig 2:**
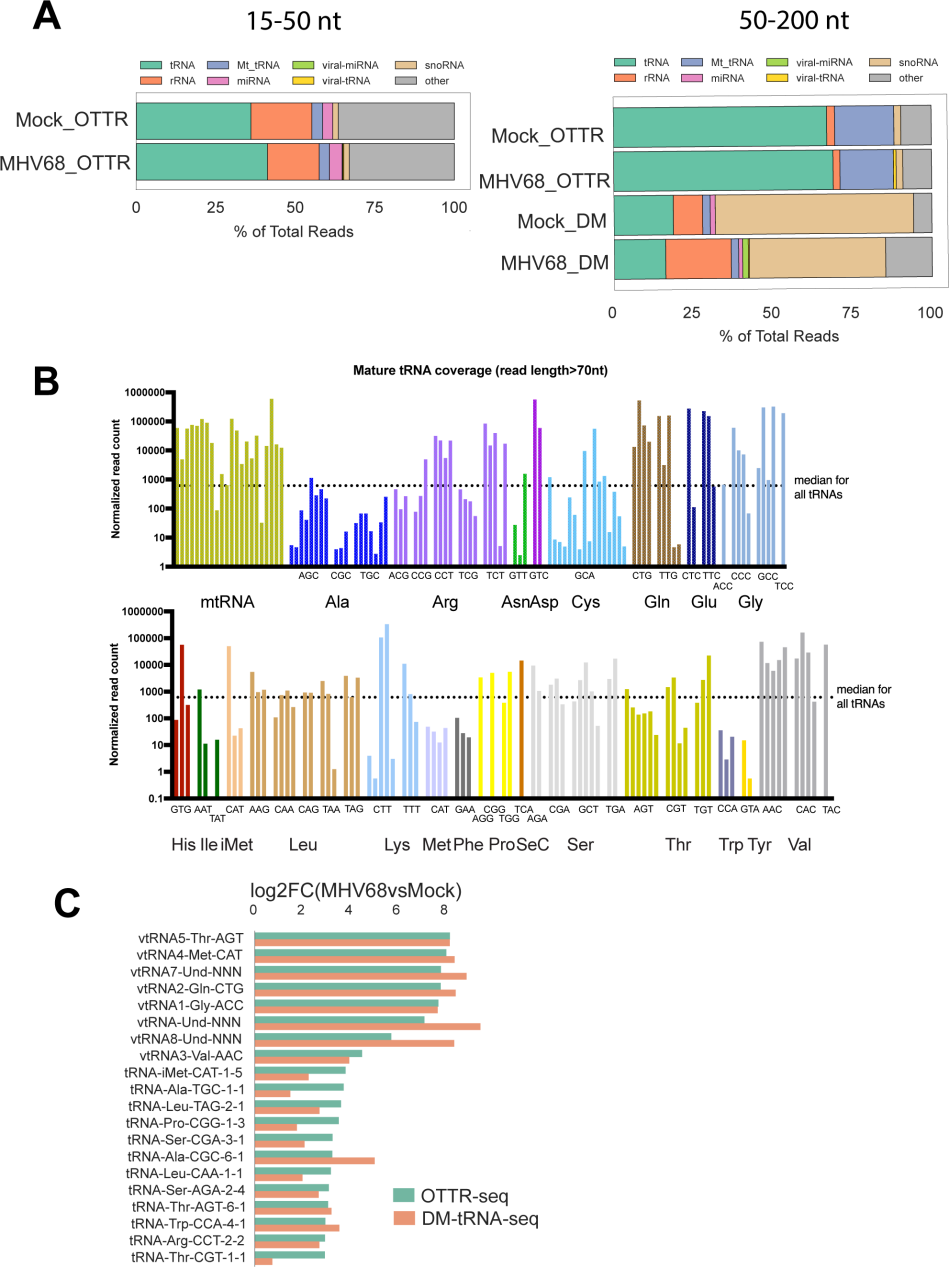
OTTR-seq robustly captures host tRNAs. (**A**) Distribution of reads mapping to various small RNA classes using OTTR-seq (OTTR) or DM-tRNA-seq (DM) prepared from mock- and MHV68-infected mouse fibroblasts at an MOI = 5 for 24 h. (**B**) Normalized read counts for host tRNA isodecoders detected in mock-infected libraries using OTTR-seq. The dotted line marks the median normalized read count for all tRNA isodecoders as reference. (**C**) Log2(fold change) values between MHV68-infected (MR) versus mock infection from OTTR-seq (green) or DM-tRNA-seq (orange) are consistent for viral-tRNAs (“virtRNA” prefix) and host pre-tRNAs.

More than 60% of the OTTR reads in the 50–200 nt size class were from tRNAs (green) (Fig. 2A, right, top two bars). In comparison, our previous DM-tRNA-seq library contained threefold fewer reads mapping to tRNAs (20%) and was instead enriched for snoRNAs (tan) at ~50% of mapped reads (6) (Fig. 2A, right bottom two bars). The composition of the 15–50 nt size class library using OTTR was derived from a variety of sources, including tRFs (green), which comprised the largest percentage at 30–40%, but also included sequences mapping to rRNAs (orange), miRNAs (pink), and snoRNAs (tan). We also assessed how well OTTR-tRAX performed on full-length mature tRNAs. To assess the levels of full-length mature tRNAs represented in our OTTR libraries, we compared normalized read counts of each tRNA isodecoder using a read-length cutoff of greater than 70 nucleotides to analyze full-length tRNA transcripts (Fig. 2B). Most isodecoders had at least one representative with expression over the median read count of all measured tRNAs (horizontal dotted line), indicating a diverse pool of tRNA species was captured. Importantly, OTTR-tRAX was robust enough to recapitulate the upregulation of viral and host pre-tRNA species during MHV68 infection we reported prior (Fig. 2C) (6).

### MHV68 infection causes host shutoff-dependent changes in tRNA expression

MHV68-induced “host shutoff,” in which cellular mRNA is endonucleolytically cleaved by the viral endonuclease muSOX (ORF37), contributes to the accumulation of pre-tRNAs in infected cells (4). Importantly, muSOX does not cleave reporter transcripts made by RNA polymerase I or III (29), arguing against the possibility of cleavage of tRNAs by muSOX. While muSOX cannot be deleted from the genome due to its essential function in genome replication, MHV68-R433I contains a single point mutation in muSOX with diminished mRNA endonucleolytic activity without impacting genome replication (20). Infection with MHV68-R443I lowers the levels of host pre-tRNA-Tyr and -Leu compared to mutant revertant, MHV68-MR, as measured by RT-qPCR (4), supporting the idea that muSOX is involved in stabilizing pre-tRNAs in infected cells. We previously hypothesized that global mRNA degradation during host shutoff depletes the cell of post-transcriptional tRNA processing factors required for pre-tRNA maturation and turnover, extending the half-life of pre-tRNAs (4, 30). Taken together, these data suggest that muSOX activity indirectly contributes to pre-tRNA accumulation during infection.

**Fig 3:**
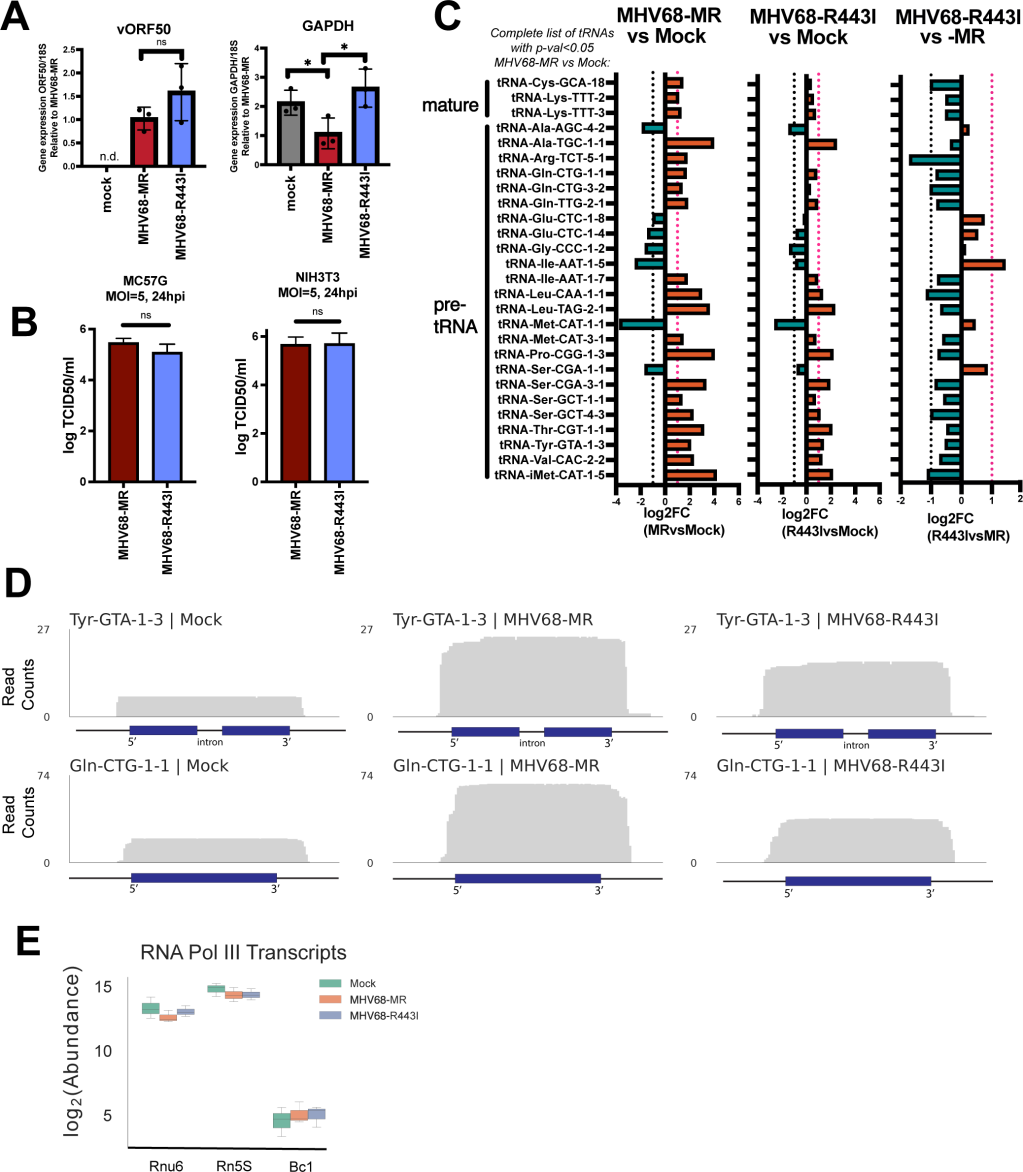
Pre-tRNA accumulation is dependent on MHV68-induced host shutoff. (**A**) RT-qPCR was performed using total RNA from mock-, MHV68-MR, or MHV68-R443I-infected MC57Gs (MOI = 5 for 24 h) to detect viral ORF50 (vORF50) and host GAPDH transcripts. Data depict means ± SD from three independent experiments relative to the MHV68-MR sample, with *P* values calculated using raw ΔCt values and unpaired *t*-test. (**B**) Supernatants from MHV68-MR and MHV68-R443I-infected MC57Gs (MOI = 5 for 24 h) were titered by TCID_50_. (**C**) The complete set of significantly differentially expressed host mature and premature tRNAs upon MHV68-MR infection (*P* < 0.05; MHV68-MR vs Mock) are plotted alongside their log2(fold change) values. Log2(fold change) values for MHV68-MR-infected versus mock (left) are graphed alongside the corresponding log2FC for MHV68-R443I versus mock (middle) and MHV68-R443I versus MHV68-MR (right). Vertical dotted lines indicate a fold change of >2 or <2. (**D**) Normalized read coverage (5′ → 3′) from mock (left), MHV68-MR (middle), or MHV68-R443I (right) across the Tyr-GTA-1-3 (top) and Gln-CTG-1-1 (bottom) tRNA gene loci. Blue boxes correspond to the gene body of the tRNA, while the horizontal black lines correspond to the 30 bp upstream, 30 bp downstream, and/or intronic regions. (**E**) Box plot showing the log2(abundance) of three RNA polymerase III transcripts in our sequenced size class (50–200 nt) colored by infection conditions.

We used OTTR-tRAX to extensively profile pre-tRNAs differentially affected during infection with MHV68-R443I, as well as to establish a benchmark of OTTR-tRAX performance under experimental conditions known to induce differential tRNA expression. MC57G mouse fibroblasts were mock-infected or infected with MHV68-R443I or MHV68-MR virus at an MOI = 5 (titer determined by TCID50; see Materials and Methods) for 24 h in biological triplicate. Notably, the MHV68-R443I virus is reported to have identical replication kinetics to MHV68-MR in cultured fibroblasts (20). We first confirmed similar levels of viral gene expression with MHV68-R443I or MHV68-MR by measuring the level of viral immediate early transcript, ORF50, expressed at 24 hpi in MC57Gs (Fig. 3A). Host shut-off activity was assessed by measuring the level of the host transcript, *Gapdh*. As expected, MHV68-MR infection leads to decreased *Gapdh* expression, while MHV68-R443I restored expression to that observed in mock-infected cells (Fig. 3A). We confirmed equivalent viral titer for MHV68-MR and -R443I after 24 hs of infection in both MC57Gs and NIH 3T3s, confirming the reported lack of replication defect for MHV68-R443I in two different fibroblast cell lines (Fig. 3B). We also confirmed the viability of cells following these infection conditions and did not detect significant LDH release or dead cells by FACS (Fig. S1). Mock-infected, MHV68-MR-infected, and MHV68-R443I RNA samples from MC57Gs were processed using OTTR-tRAX (Fig. 3C). All differentially expressed (*P* < 0.05) pre- or mature tRNAs from the 50–200 nt size class upon MHV68-MR infection compared to mock are depicted in Fig. 3C (MHV68-MR vs Mock). The majority of significantly differentially expressed transcripts upon MHV68-MR infection are premature tRNA sequences, as seen previously using DM-tRNA-seq (4). MHV68-R443I infection increased pre-tRNA expression but to a lesser degree, likely due to residual host shutoff activity or additional contributing factors, including enhanced RNA Pol III recruitment as reported prior (4) (Fig. 3C, MHV68-R443I vs Mock). However, the majority of pre-tRNAs exhibited decreased expression in MHV68-R443I compared to MHV68-MR (Fig. 3B, MHV68-R443I vs MHV68-MR), confirming that pre-tRNA accumulation is enhanced by muSOX activity. Read coverage plots of pre-tRNA-Tyr-GTA-1-3 and pre-tRNA-Gln-CTG-1-1 illustrate the reduced pre-tRNA expression with MHV68-R443I versus MHV68-MR virus (Fig. 3D). There were no differences in the lengths of the 5′ leaders or 3′ trailers of pre-tRNAs between mock- and MHV68-infected cells, suggesting that these transcripts correspond to nascent pre-tRNAs prior to trimming or splicing. Differential upregulation is specific to a subset of pre-tRNAs (Fig. 3C), as other non-coding RNAs made by RNA polymerase III do not increase in abundance during infection with MHV68-MR or -R443I (Fig. 3E). Overall, OTTR-tRAX reveals global changes in pre-tRNA abundance that are partially dependent on the host shutoff functionality of MHV68 muSOX.

### tRFs are generated during MHV68 infection and are dependent on host shutoff

**Fig 4:**
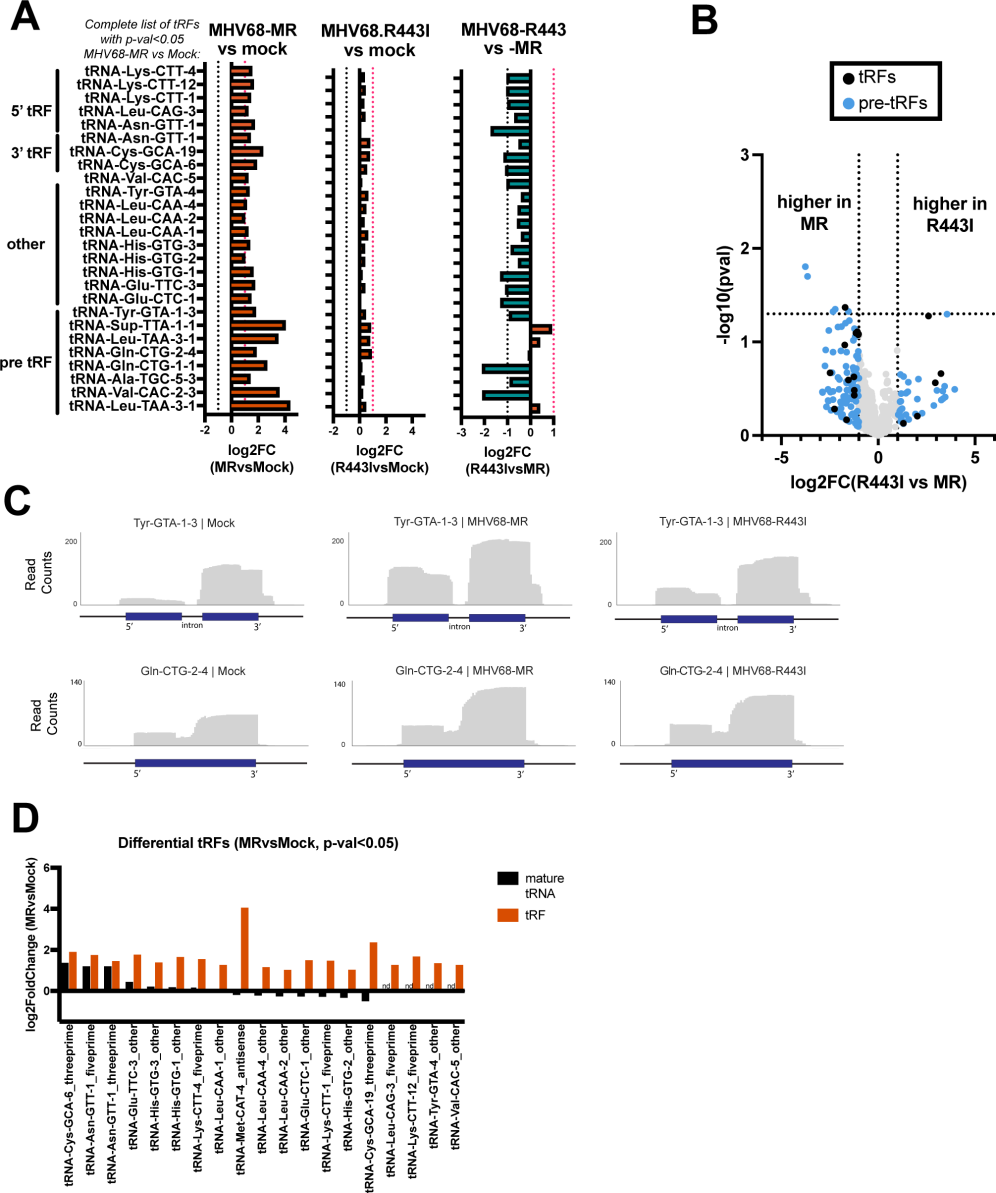
tRNA fragments (tRFs) are induced in a host shutoff-dependent manner. (**A**) The complete set of significantly differentially expressed host tRFs (host cytosolic 5′tRFs, 3′tRFs, internal tRFs, and premature-derived tRFs) upon MHV68-MR infection (*P* < 0.05; MHV68-MR vs Mock) are plotted alongside log2(fold change) values. Log2(fold change) values of MHV68-MR versus mock (left) are graphed alongside the corresponding log2FC for MHV68-R443I versus mock (middle), and MHV68-R443I versus MHV68-MR (right). Vertical dotted lines indicate a fold change of >2 or <2. (**B**) Volcano plot of log2(FC) values for tRFs from MHV68-MR versus MHV68-R443I infections, plotted against −log10(*P* value). tRFs up- or downregulated more than twofold are colored blue for pre-tRFs or black for tRFs generated from mature tRNAs. (**C**) Normalized read coverage (5′ → 3′) from mock (left), MHV68-MR (middle), or MHV68-R443I (right) across tRNA gene loci. Blue boxes correspond to the gene body of the tRNA, while the horizontal black lines correspond to the 30 bp upstream, 30 bp downstream, and/or intronic regions. (**D**) Significantly differentially expressed host tRFs that appear to be derived from mature tRNAs (5′tRFs, 3′tRFs, and internal tRFs) upon MHV68-MR infection (*P* < 0.05; MHV68-MR vs Mock) are plotted. The log2(fold change) for both the parental mature tRNA (black bars) and the tRFs (orange bars) are graphed for comparison. nd = not detected in sequence run. There is no correlation between changes in mature tRNA and their cognate tRFs.

We next used OTTR-tRAX to examine global changes in tRF expression during infection, as this has not been explored during DNA virus infection. OTTR- tRAX analysis of the 15–50 nt size class confirmed the production of tRFs in MHV68-infected cells (Fig. 4A, left, MHV68-MR vs mock infection, all tRFs with *P* < 0.05 are depicted; Fig. S2 to S4). Based on tRAX parameters, tRFs are binned according to the best match source sequence, with priority given to mature tRNA reference sequences, which were curated for the processed mature tRNA sequence complete with 3′ CCA tails (22). tRAX assigns these tRFs as “5′ tRFs” if they map within 5 nt of the annotated 5′ end of the corresponding mature tRNA, “3′ tRFs” if they map within 5 nt of the annotated 3′ end of the corresponding mature tRNA, or “other” if they do not meet these parameters. In contrast, reads that contain leader, trailer, or intronic sequence do not map to the mature tRNA reference and instead map to genomic reference sequences and were binned as “pre-tRFs.” For many tRNA genes, there were distinct 5′ and 3′ pre-tRFs produced from the same transcript (Fig. 4C). For example, pre-tRFs from the intron-containing tRNA-Tyr-GTA-1-3 gene originated from both the 5′ and 3′ exons, with extensions into nearby leader, intron, or trailer sequence. Pre-tRFs were also observed from intron-less tRNA genes, such as tRNA-Gln-CTG (Fig. 4C). However, pre-tRFs from Gln-CTG tRNA genes had more heterogeneous ends compared to pre-tRFs from Tyr-GTA. Among the other tRFs upregulated in infected fibroblasts were 5′, 3′, and internally derived tRFs (Fig. S2 to S4). Almost all of the significantly upregulated 5′ tRFs had distinct 3′ ends, which resided in or around the D-loop (Fig. S2). In contrast, 3′ and internally derived tRFs exhibited more heterogeneous ends, suggestive of ongoing exonucleolytic degradation (Fig. S3 and S4). In some cases, the tRFs could be mapped uniquely to a specific tRNA locus of origin (e.g., 3′ tRF-Asn-GTT-1 in Fig. S3 or int-tRF-His-GTG-1 in Fig. S4, purple reads). Previous studies have suggested that increased tRF levels do not typically correlate with a decrease in mature tRNA substrate (31). We echo these findings here, as differentially expressed tRFs are derived from mature tRNAs that exhibit a wide range of expression changes in response to MHV68 infection (Fig. 4D). tRFs as a whole showed higher expression in MHV68-MR compared to MHV68-R443I (Fig. 4A and B; Fig. S2 to S4), with a few exceptions (see Glu-CTC-5 in Fig. S2). Overall, the dependence on muSOX activity was more striking for tRFs than observed for pre-tRNAs, as assessed by the difference in log2FC values presented in Fig. 3B vs. Fig. 4A. These results illustrate that MHV68 triggers tRF production, and that this phenomenon is dependent on host shutoff activity by MHV68 muSOX.

### Viral tRNAs are modified and undergo cleavage into tRFs

**Fig 5:**
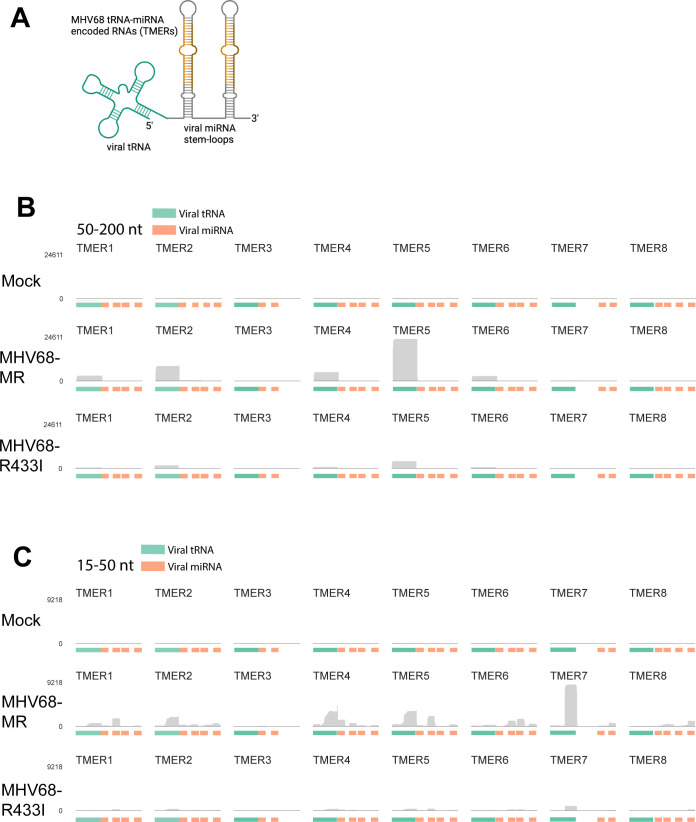
Viral TMERs are induced in a host-shutoff-dependent manner. (**A**) Schematic of a viral TMER highlighting the 5′ virtRNA and 3′ miRNA regions. (**B**) Normalized read coverage across the eight viral TMERs encoded within the MHV68 genome from the 50–200 nt size selected libraries for mock (top), MHV68-MR (middle), and MHV68-R433I (bottom). (**C**) Normalized read coverage across TMERs from the 15–50 nt size selected libraries for mock (top), MHV68-MR (middle), and MHV68-R433I (bottom). Color bars below indicate either a viral-tRNA feature (green) or a viral-miRNA feature (orange).

We expected OTTR-tRAX might be a useful tool to examine changes in viral TMER and TMER-derived small RNAs during MHV68 infection. There are eight encoded TMERs in the MHV68 genome, and TMER transcripts play an important role in pathogenesis and latency establishment during MHV68 infection in mice (13, 14). Transcription of the TMERs by RNA polymerase III results in a hybrid RNA molecule consisting of a viral tRNA (virtRNA) fused to one or two miRNA SLs (Fig. 5A). We use the “virtRNA” nomenclature here instead of the previously used “vtRNA” to avoid confusion with host vault RNAs. Processing of TMERs involves cleavage by the host enzyme ELAC2, normally responsible for removing the 3′ trailer during host tRNA maturation, to separate the viral miRNA SLs from the virtRNA (16).

DICER then further processes the viral miRNAs for use by host silencing machinery. There is evidence that virtRNAs undergo maturation in the form of post-transcriptional CCA addition at the 3′ ends (32), which is a required step in host tRNA maturation to form the site of amino acid attachment. However, virtRNAs are not detectably charged with amino acids, at least for the virtRNAs previously tested (virtRNA3–6) (32). We mapped OTTR-tRAX reads from both small and large-size classes to MHV68 TMER loci (Fig. 5B and C). Our size selection at <200 nt excluded the majority of full-length TMER transcripts (~200–250 nt); however, we detected robust expression of virtRNAs from TMERs 1, 2, 4, 5, and 6 (Fig. 5B). A specific isoform of TMER4 consisting of virtRNA4 plus SL1 (vtRNA-SL1) (33) was recently reported to drive latency establishment *in vivo*. While the size of vtRNA-SL1 (~155 nt) should not prevent inclusion in our sequencing libraries, this ncRNA comprised less than 0.5% of virtRNA4-containing reads, suggesting that vtRNA-SL1 is not expressed during infection of MC57G fibroblasts. We were able to detect differential expression of viral miRNAs during infection in fibroblasts (Fig. 5C; detailed view in Fig. S5). The three most abundant viral miRNAs included mghv-miR-M1-1-3p (TMER1), mghv-miR-M1-7-3p (TMER5), and mghv-miR-8-5p (TMER6). Interestingly, we detected 3′ fragments made from seven out of eight virtRNAs (TMER1–7), and in some cases, these 3′ fragments were more abundant than the co-transcribed viral miRNAs (TMER2, 3, 4, 5, and 7). TMER7 produced a highly abundant 3′ tRF, yet the virtRNA from this locus was not detected (compare Fig. 5B and C). MHV68-R443I infection resulted in globally reduced expression of both virtRNAs and viral miRNAs compared to MHV68-MR, supporting previous microarray analysis (30). Similarly, virtRNA fragment expression was also reduced. Altogether, our analysis revealed that virtRNAs are cleaved to produce shorter fragments, similar to host tRNAs.

### tRFs derived from pre-tRNAs are generated during MHV68 infection

We next explored the potential biogenesis mechanisms of host tRFs. We noted that approximately one-third of tRFs produced in MHV68-MR-infected cells were derived from pre-tRNAs based on retained leader, trailer, or intronic sequences, leading us to hypothesize that upregulated pre-tRNAs are a major source of tRFs during infection. This was supported by our observation that MHV68-MR exhibited higher pre-tRNA accumulation and higher tRF expression, while MHV68-R443I showed lower pre-tRNA accumulation and lower tRF expression (Fig. 4A and B). We reasoned that if tRFs are sourced from accumulating pre-tRNAs during infection, we would see a positive correlation between parental pre-tRNA and pre-tRF levels. We performed correlation analysis comparing log2FC(MRvsMock) for pre-tRFs versus the parental pre-tRNA (Fig. S6) to assess the correlation between these two transcript classes. There was a weak positive correlation between isogenic pre-tRNAs and pre-tRFs, with a Pearson’s correlation coefficient (*r*) of 0.33 (*P* value ≤ 0.001) and an *r*^2^ value of 0.11. This is not unexpected considering that steady-state levels of pre-tRNAs and tRFs are dependent on stability features, including modification status and/or protein binding partners, which might vary wildly between the two classes of RNAs. However, when we looked at each tRNA family individually, there were several tRNA families with strong, statistically significant positive correlation, including -Ala, -Asp, -Leu-, -Lys, and -Tyr (Fig. S6). Additionally, there was a strong positive correlation when we looked specifically at those tRNAs encoding introns (Fig. S6; *r* = 0.77; *P* value < 0.0001; *r*^2^ = 0.60), which we posit is due to stronger mapping accuracy for pre-tRNAs and pre-tRFs. Taken together, we suspect that OTTR-tRAX underestimates pre-tRNA-derived tRFs, as internally derived tRFs lacking these identifying pre-tRNA features would be identical whether sourced from pre- or mature tRNAs, with tRAX prioritizing sequence mappings to mature tRNA reference sequences (22). Regardless, there is a strong signature supporting that pre-tRNAs are substrates for endonucleolytic cleavage during MHV68 infection. Taken together, our data reveal a mechanistic link between pre-tRNA accumulation and tRF production.

**Fig 6:**
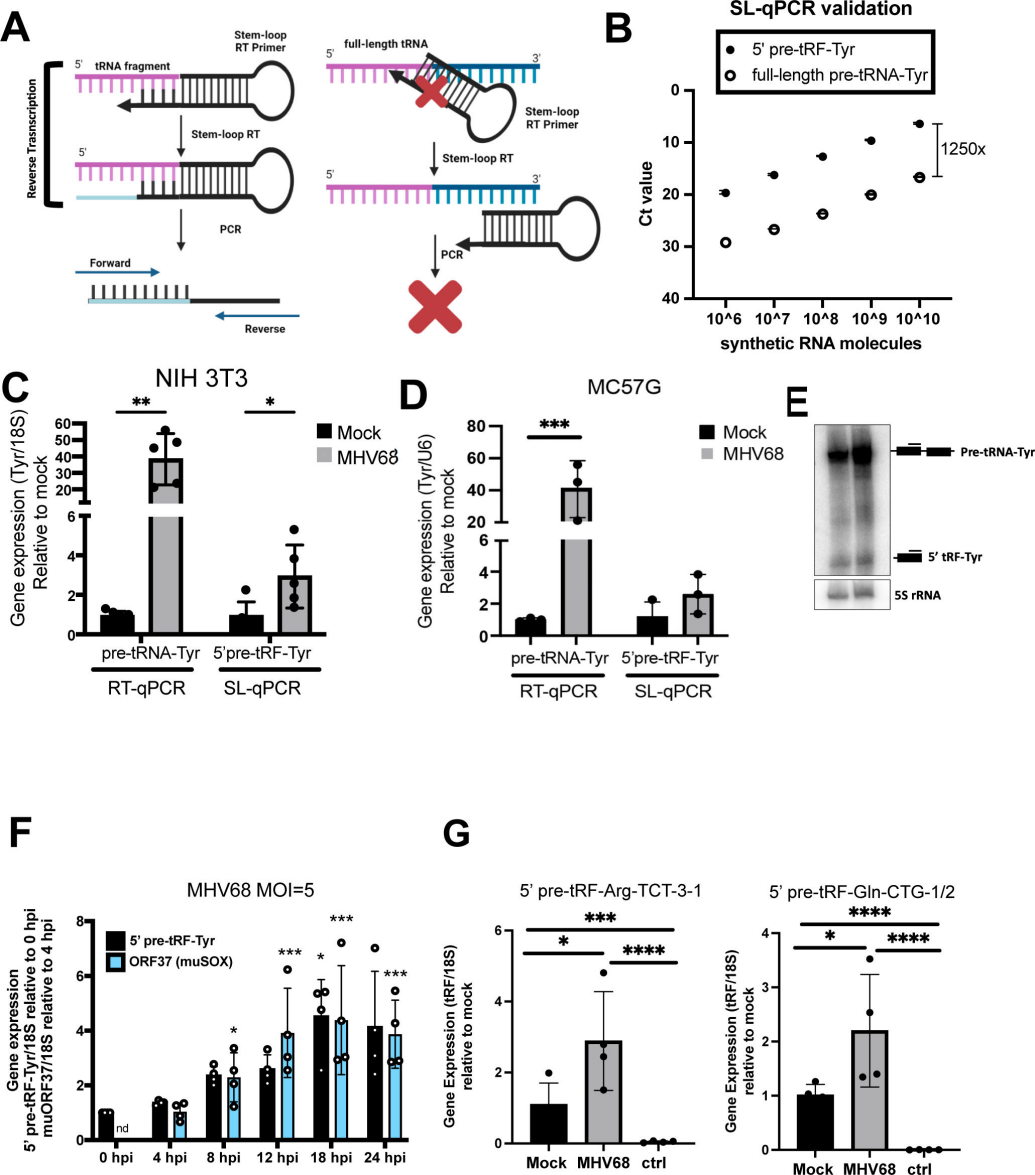
Stem-loop (SL) qPCR detection of 5′ tRFs. (**A**) Schematic illustrating the specificity of the SL RT primer for 5′ pre-tRF versus full-length parental tRNA. (**B**) Synthetic full-length pre-tRNA-Tyr or 5′ pre-tRF-Tyr were used in known quantities for SL-qPCR using Tyr forward and SL reverse primers. Raw Ct values are shown. (C and D) Standard- and SL-qPCR was performed using total RNA from mock- or MHV68-infected (MOI = 5 for 24 h) NIH 3T3s (**C**) or MC57Gs (**D**) to detect the full-length pre-tRNA-Tyr or 5′ pre-tRF-Tyr, respectively. 18S or U6 primers were used as endogenous controls. Data depict means ± SD from three to five independent experiments relative to the mock sample. (**E**) Northern blot using 5′ exon Tyr probes was performed using mock- or MHV68-infected (MOI = 5 for 24 h) MC57G RNA samples. (**F**) SL-qPCR was performed from four independent experiments to detect 5′ pre-tRF-Tyr produced in NIH 3T3 during infection (MOI = 5 for 24 h). Data depict means ± SD relative to the 0 h time point of each experiment. (**G**) SL-qPCR was performed from four independent experiments to detect 5′ tRF-Arg-TCT and -Gln-CTG using mock- or MHV68-infected (MOI = 5 for 24 h) NIH 3T3 RNA samples. The sample labeled “ctrl” was generated with RNA reverse transcribed with a SL RT primer for an unrelated tRF species. tDRnamer (21) nomenclature for amplified tRFs is reported in Materials and Methods. All *P* values were calculated using raw ΔCt values and *t*-test. ns = *P* > 0.05; *P ≤ 0.05; **P ≤ 0.01; ***P ≤ 0.001.

### Validation of 5′ tRF production using SL RT-qPCR

Next, we applied a method to detect 5′ pre-tRFs in response to MHV68 infection using an orthologous approach called SL RT-qPCR. SL-qPCR allows the selective amplification of 5′ cleavage products versus their full-length counterparts (34) (Fig. 6A). Briefly, SL-RT primers are designed to be specific for the 3′ end of the cleavage product. This SL-RT primer should preferentially bind to the cleavage product, not the precursor, as the precursor has a different 3′ end and additionally cannot be internally primed due to the steric hindrance with the SL-RT primer. We designed SL-RT primers for the upregulated 5′ pre-tRF-Tyr identified by OTTR-tRAX, as we had also validated its expression by northern blot (4). We assessed the specificity of SL-qPCR for detecting 5′ pre-tRF-Tyr rather than full-length pre-tRNA-Tyr by using synthetic RNAs (Fig. 6B). SL-qPCR was performed using a dilution series of equimolecular full-length and fragment synthetic RNAs as templates and confirmed >1,000-fold specificity for fragment detection. We then applied the SL-qPCR assay for 5′ pre-tRF-Tyr to cellular RNA from NIH 3T3 (Fig. 6C) and MC57G (Fig. 6D) cells ± MHV68 infection. We measured full-length pre-tRNA-Tyr using standard RT-qPCR and a reverse qPCR primer specific for the intron (absent in 5′ pre-tRF-Tyr) alongside as a control (4). There was a 2.5-fold increase in 5′ pre-tRF-Tyr levels upon infection, which was similar between MC57G and NIH 3T3, and to that observed by northern blot done in parallel (Fig. 6C through E). Using this SL-qPCR assay, we next assessed the induction of 5′ pre-tRF-Tyr over the course of a 24-h infection at an MOI = 5 (Fig. 6F). We observed a peak of 5′ pre-tRNA-Tyr at 18 hpi, with 5′ pre-tRF-Tyr showing patterns of a biphasic induction with a twofold increase evident between 8 and 12 hpi, and a fourfold total increase evident by 18 hpi. By comparison, ORF37 (muSOX) reaches peak expression at 12 hpi. These data are supportive of a scenario where muSOX (peaking at 12 hpi) induces 5′ pre-tRF-Tyr expression (peaking at 18 hpi). We designed SL-qPCR assays for other 5′ tRFs, including 5′ pre-tRF-Arg-TCT-3-1 and 5′ tRF-Gln-CTG-1/2, and confirmed their upregulation during MHV68 infection (Fig. 6G).

### 5′ pre-tRF-Tyr expression is dependent on tRNA splicing factors

**Fig 7:**
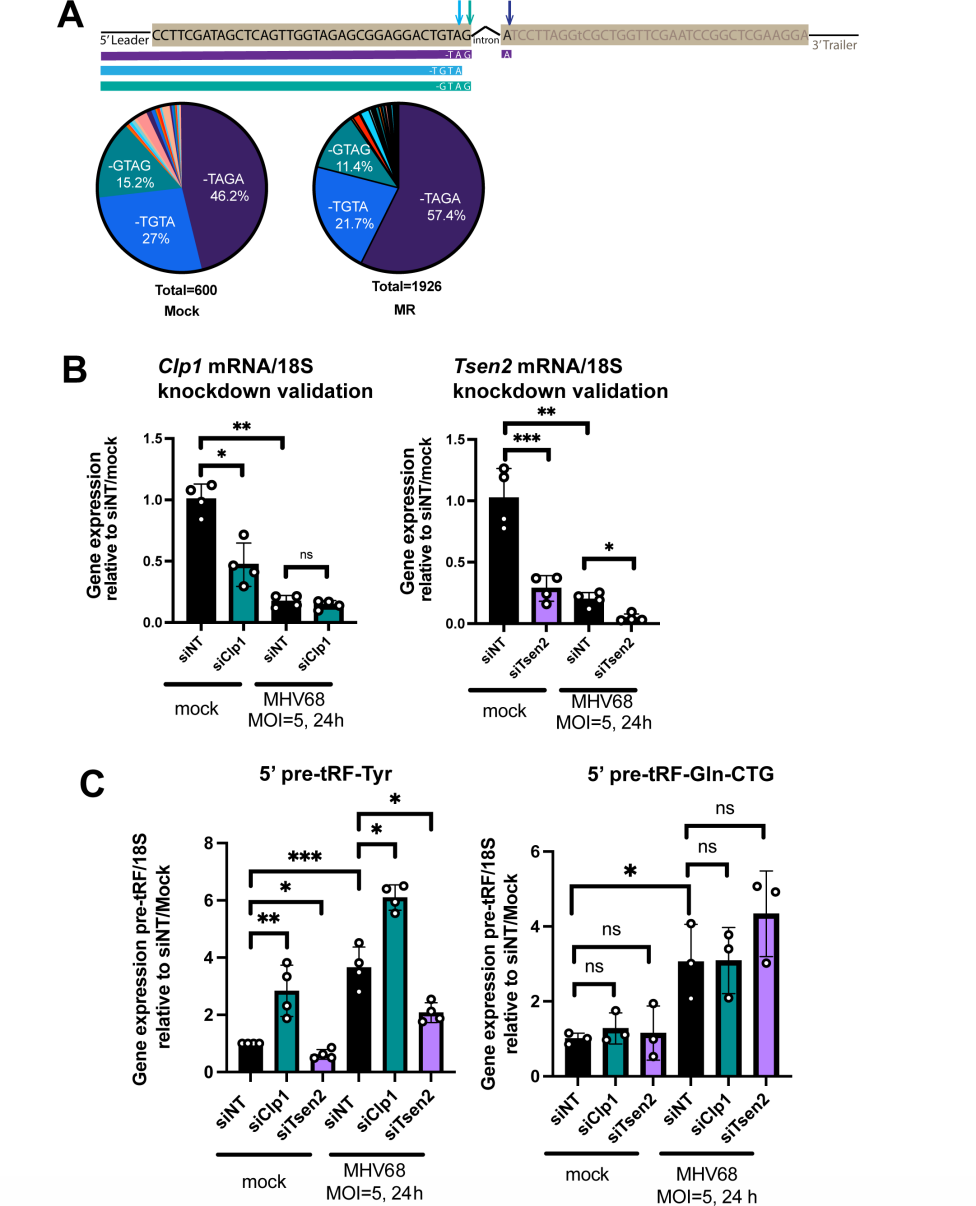
The tRNA splicing factors TSEN2 and CLP1 modulate 5′ pre-tRF-Tyr expression during MHV68 infection. (**A**) Schematic depicting a pre-tRNA-Tyr transcript (top line) and top three most abundant 5′ pre-tRF-Tyr transcripts detected (purple, blue, and teal lines). The pie charts depict the percentage of detected termini of pre-tRF-Tyr transcripts from mock- and MHV68-MR-infected MC57G fibroblasts using OTTR-tRAX. (**B**) siRNA-mediated knockdown of *Tsen2* (siTsen2) and *Clp1* (siClp1) or treatment with non-targeting siRNAs (siNT) was followed by mock or MHV68 infection at an MOI = 5 for 24 h. siRNA knockdown was confirmed using RT-qPCR using *Clp1*, *Tsen2*, and *18S*-specific primers. (**C**) SL-qPCR was used to measure 5′ pre-tRF-Tyr upon siRNA treatment. Data from RT-qPCR and SL-qPCR experiments are depicted as mean ± SD from four independent experiments relative to the siNT/mock sample, with *P* values calculated using raw ΔCt values and paired *t*-test. (**D**) Viral titer of supernatants was measured by TCID_50_ from three independent experiments, with *P* values calculated by one-way ANOVA. ns = *P* > 0.05; *P ≤ 0.05; **P ≤ 0.01; ***P ≤ 0.001.

We noted that the 3′ end of 5′ pre-tRFs made from intron-containing tRNA genes (such as pre-tRNA-Tyr) aligned with the splice junction (Fig. 4C), suggesting that tRNA splicing may occur prior to or during pre-tRF biogenesis. Additionally, 5′ pre-tRF-Tyr upregulation was previously reported in fibroblasts and tissue extracted from kinase-dead *Clp1^K/K^* mice (35). CLP1 kinase associates with the tRNA splicing complex (TSEN2, 15, 34, 54) and negatively regulates tRNA splicing (36–38). We observed that the most abundant terminal ends of 5′ pre-tRF-Tyr (-TAGA, -TGTA, and -GTAG) were downstream of the anticodon (GTA) and bordered the terminal nucleotide of the 5′ tRNA-Tyr exon (Fig. 7A). The 3′ end -TAGA was the most abundant transcript termini observed in mock-infected cells (46.2%) and composed a slightly higher percentage in MHV68-infected cells (57.4%). Though 5′ leader nucleotides are present on 5′ pre-tRF-Tyr, the terminal -TAGA most likely results from cleavage of the anticodon loop post-splicing, as the terminus spans to the first nucleotide of the 3′ exon. This might suggest that intron removal can occur prior to trimming of the 5′ leader, which has not yet been described in mammalian cells, or perhaps solely is a byproduct cleaved from an aberrant tRNA precursor. In contrast, the -TGTA and -GTAG termini may potentially represent splicing intermediates.

The tRNA splicing endonuclease, TSEN2, was previously shown to be involved in 5′ pre-tRNA-Tyr production in *Clp1^K/K^* mouse embryonic fibroblasts (35), and so we tested whether the same was true during MHV68 infection. To test whether 5′ pre-tRF-Tyr was sensitive to decreased expression of tRNA splicing factors, we used siRNAs to knock down the expression of both *Clp1* and *Tsen2* in NIH 3T3s (Fig. 7B). We validated knockdown of *Clp1* and *Tsen2* mRNA expression by RT-qPCR, as we were unable to obtain commercial antibodies of sufficient specificity (Fig. 7B). Both knockdowns reduced expression of their target mRNA in mock-infected cells. Knockdown did not reach statistical significance for *Clp1* in MHV68-infected cells; however, the level of *Clp1* and *Tsen2* mRNAs, like most host transcripts, is diminished due to host shutoff during infection and approached the limit of detection (Fig. S7A). We confirmed that cells remained viable after knockdown of *Tsen2* and *Clp1* followed by MHV68-MR infection (Fig. S7B and C). *Clp1* knockdown was associated with enhanced 5′ pre-tRF-Tyr expression in both mock- and MHV68-infected cells, confirming that CLP1 negatively regulates the production of 5′ pre-tRF-Tyr in both contexts (Fig. 7C). In contrast, *Tsen2* knockdown decreased 5′ pre-tRF-Tyr levels, suggesting that TSEN2 contributes to the accumulation of 5′ pre-tRF-Tyr in mock- and MHV68-infected cells, similar to *Clp1^K/K^* MEFs (35). To test whether the generation of pre-tRF fragments by Tsen2 was specific to intron-containing pre-tRNAs, we also performed SL-qPCR for 5′ pre-tRF-Gln-CTG derived from intron-less pre-tRNA-Gln-CTG genes. Though 5′ pre-tRF-Gln-CTG is enhanced by infection upon treatment with non-targeting siRNAs, as expected, the knockdown of Clp1 and Tsen2 has no effect on 5′ pre-tRF-Gln-CTG compared to the non-targeting control (Fig. 7C). Taken together, these data suggest that while tRNA splicing machinery can trigger pre-tRFs from intron-containing pre-tRNAs, other yet-to-be identified endonucleases and/or cleavage mechanisms are involved in pre-tRF generation.

### Clp1 is required for efficient MHV68 replication

**Fig 8:**
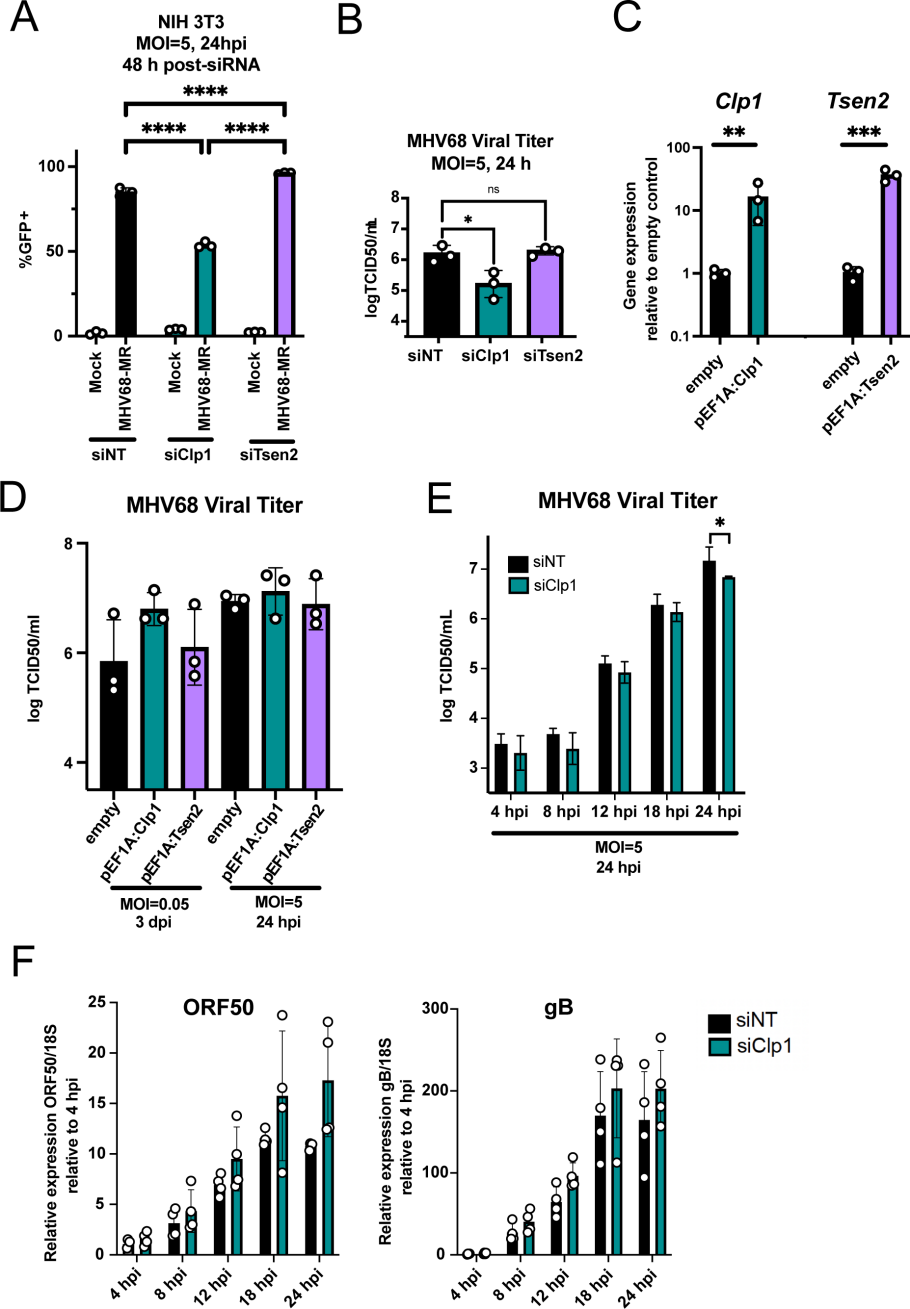
CLP1 is required for efficient MHV68 replication. (**A**) siRNA-mediated knockdown of *Tsen2* (siTsen2) and *Clp1* (siClp1) or treatment with non-targeting siRNAs (siNT) was followed by mock or MHV68-MR infection at an MOI = 5 for 24 h. Cells were analyzed for GFP expression by flow cytometry. (**B**) Supernatants from the conditions in (**A**) were titered by TCID_50_. (**C**) Stable cell lines expressing pEF1A:empty (empty), pEF1A:*Clp1*, or pEF1A:*Tsen2* were validated by RT-qPCR using *Clp1*, *Tsen2*, and *18S*-specific primers. (**D**) Stable cell lines expressing pmCherry (empty), pEF1A:*Clp1*, or pEF1A:*Tsen2* were infected at both low (MOI = 0.05) and high (MOI = 5) MOIs and titered at 3 dpi or 24 hpi, respectively, by TCID_50_. (**E and F**) siRNA-mediated knockdown of *Clp1* (siClp1) or treatment with non-targeting siRNAs (siNT) was followed by MHV68-MR infection at an MOI = 5. Samples were collected at indicated time points and analyzed by TCID_50_ (**E**) or RT-qPCR using viral ORF50, viral gB, and *18S*-specific primers (**F**). All *P* values were calculated using raw ΔCt or TCID_50_/mL values and one-way ANOVAs or unpaired *t*-test. ns = *P* > 0.05; *P ≤ 0.05; **P ≤ 0.01; ***P ≤ 0.001.

Next, we performed a single round of MHV68 replication (MOI = 5) in cells treated with non-targeting, *Clp1*, or *Tsen2*-targeting siRNAs. We measured both the percentage of infected (GFP^+^) cells in the siRNA-treated culture, as well as infectivity in the associated cell media by TCID_50_ (Fig. 8A and B). *Clp1* knockdown resulted in decreased GFP positivity of the culture (Fig. 8A) and an eightfold decrease in MHV68 titer (Fig. 8B) compared to the non-targeting control. *Tsen2* knockdown showed slightly higher GFP positivity (Fig. 8A), but did not yield differences in released infectious virions (Fig. 8B). These data suggest that tRNA splicing factors can impact viral gene expression during infection, with CLP1 required for the release of infectious virus. We next overexpressed *Clp1* and *Tsen2* by transducing NIH 3T3 cells with lentiviral vectors carrying *Clp1* and *Tsen2* fused to an EF1A promoter. We confirmed that *Clp1* and *Tsen2* mRNAs were overexpressed by ~16-fold and ~36-fold, respectively, in these stable cell lines compared to a cell line transduced with an empty lentiviral vector (Fig. 8C). However, there was no significant difference in MHV68 titers produced from either pEF1A:*Clp1* or pEF1A:*Tsen2* cell lines, though two of the three biological replicates suggested enhanced titers from the pEF1A:*Clp1* cell line following a low MOI infection (Fig. 8D). This result suggests that CLP1 supports MHV68 replication, but that overexpression of neither CLP1 nor TSEN2 significantly affects MHV68 replication. To define the stage of replication impacted by *Clp1* knockdown, we performed a time course experiment following MHV68 infection at an MOI = 5 (Fig. 8E). *Clp1* knockdown subtly decreased titers across the time course (though not statistically significant at time points between 0 and 18 hpi) and resulted in decreased MHV68 titer at 24 hpi. To determine if the drop in infectious titer correlated with decreased viral gene expression, we performed RT-qPCR to measure ORF50 and the late gene, gB (Fig. 8F). Both viral transcripts were similar at each time point upon Clp1 knockdown, suggesting that Clp1 supports viral replication at a stage post late-gene transcription. Taken together, our data demonstrate that CLP1 supports late-stage MHV68 replication. Though CLP1 is implicated in tRNA splicing, whether the *Clp1*-dependent effect on MHV68 replication is directly from altered tRNA splicing activity should be tested in future studies.

## DISCUSSION

While there have been reports of tRFs produced in response to RNA virus infection (2, 5, 18); here, we show that DNA viruses can also induce host tRNA cleavage. By combining OTTR-seq for library production with the bioinformatic software tRAX, we conducted the first in-depth analysis of tRNA and tRF expression during MHV68 infection. OTTR-tRAX offers a significant improvement over currently used tRNA sequencing strategies, as it (i) utilizes a novel bioengineered RT that effectively copies modified tRNAs/tRFs and (ii) minimizes sequencing artifacts. During the publication of this work, an independent study confirmed the superior utility of OTTR to sequence tRNAs and tRFs over other small RNA library preparations (39). OTTR-tRAX facilitated the discovery that a variety of tRFs are upregulated in response to MHV68, with the majority derived from pre-tRNAs that accumulate during infection. Based on MHV68-MR and MHV68-R443I sequencing, we note a correlation between elevated tRF levels and the parental transcript of origin for numerous tRNA families. As viruses belonging to each of the three herpesvirus subfamilies (alpha, HSV-1; beta, HCMV; and gamma, MHV68) have now been reported to induce tRNA transcription (4, 40, 41), it will be interesting to examine if pre-tRNA cleavage is also a universal response to herpesvirus infection.

Our data suggest that the accumulation of pre-tRNA transcripts during MHV68 infection drives the production of tRFs. Approximately one-third of the tRFs produced retain sequences that confirm their derivation from premature tRNA substrates. Further, there is a general positive correlation between the differential expression of isogenic pre-tRNAs and their tRF products induced by MHV68. And most strikingly, we saw that decreased pre-tRNA accumulation with MHV68-R443I versus MHV68-MR resulted in decreased tRF expression. There is also reason to assume that OTTR-tRAX may underestimate the total number of pre-tRFs, given that pre-tRFs that do not retain leaders, trailers, or introns are indistinguishable from tRFs derived from mature tRNAs. Overall, it is possible that infection-induced tRFs binned as mature tRFs could in fact be derived from pre-tRNAs, and this is currently being investigated. It is possible that other tRNA mapping software and strategies that do not carefully bin pre- versus mature-sequencing reads might also report tRFs that are made from pre-tRNA substrates. There are known examples of pre-tRF formation and functionality, including a 3′ trailer-derived tRF that sequesters La/SSB to block RNA virus infection (2). Based on the abundance of tRFs made from pre-tRNAs we identified during MHV68 infection, it is intriguing to consider the possibility that tRFs produced during other infections (including herpesvirus reactivation from latency) or stress scenarios are also derived from pre-tRNAs, and that their functionality may be driven by pre-tRNA-specific sequence and hypomodification characteristics.

Viruses often encode “mimics” of host machinery to facilitate infection, and TMERs encoded by MHV68 are an intriguing example of this viral strategy. TMERs are transcribed by host RNA polymerase III and undergo maturation by host tRNA and miRNA processing factors, including Elac2 and Dicer (16). Early studies revealed that the virtRNAs are not charged (32), and thus virtRNAs have been considered as solely promoter sequences for the expression of downstream viral miRNAs (14) or other recently discovered ncRNAs (33) made from the TMER4 gene. Both viral miRNAs and the TMER4-derived ncRNA are important for latency establishment in wild-type (WT) mice and do not elicit lethal pneumonia observed following WT MHV68 infection in IFNγ-deficient mice (13–15). Here, we were able to map viral miRNAs expressed in response to MHV68 *de novo* infection in MC57G murine fibroblasts. Compared to SL RT-qPCR data from infected NIH 3T12 murine fibroblasts, there are several viral miRNAs that are highly expressed in both cell lines (e.g., mghv-miR-M1-8-5p) (14). Other viral miRNAs, including mghv-miR-M1-1-3p and -7-3p, are uniquely expressed to high levels in our data set, echoing previous work demonstrating that viral miRNA expression is cell-type dependent (14). For unclear reasons, we were unable to detect the TMER4-derived ncRNA vtRNA-SL1 (33), which might be explained by a similar cell-type dependency. Our new data also emphasize how widely the expression levels of different virtRNAs vary, with virtRNA5 being the most abundant in fibroblasts. Our finding that full-length virtRNA7 is not detected by RNA sequencing aligns with published data, as well as initial predictions based on sequence gazing that virtRNA7 encodes an intron and predicted fold that would not be conducive to proper splicing (15, 32, 42). Surprisingly, 3′ tRFs are made from TMER-derived virtRNAs as well, with greater abundance than their downstream miRNA counterparts, expanding the functionality and known coding capacity of these unique viral genes. The abundance and maturation status of virtRNAs and other TMER fragments should be explored further, including during *in vivo* infection, to expand our understanding of the functional capacity of TMERs and other viral non-coding RNAs.

Our data suggest that tRNA splicing contributes to host pre-tRF formation in mouse fibroblasts during MHV68 infection. We noticed infection-induced pre-tRFs derived from pre-tRNA-Tyr with 3′ terminal ends that matched or bordered the terminal ends of the corresponding tRNA 5′ exon. Because of this observation, we explored the possibility that these tRFs form due to dysregulated tRNA splicing events, as reported in Clp1 kinase-dead MEFs (35). During splicing of pre-tRNA-Tyr, TSEN2 cleaves at the 5′ exon-intron junction, generating an expected transcript end that was abundant in 5′ pre-tRF-Tyr reads. We found that TSEN2 activity contributes to 5′ pre-tRF-Tyr production in both mock- and MHV68-infected cells, suggesting that 5′ pre-tRF-Tyr may be a splicing intermediate. In contrast, the expression of 5′ pre-tRF-Gln-CTG, which is made from an intron-less pre-tRNA, was not affected by *Tsen2* or *Clp1* knockdown. The fact that the 5′ pre-tRF-Tyr retains its 5′ leader (4, 35) supports the idea that intron removal occurs prior to 5′ leader removal in mammalian cells (43, 44). Another intriguing possibility is that 5′ pre-tRF-Tyr reported here and elsewhere could be generated by a reported self-cleavage activity inherent in pre-tRNA-Tyr at both 5′ and 3′ splice sites (45). Inherent self-cleavage activity could explain why *Tsen2* knockdown only leads to a ~50% reduction in 5′ pre-tRF-Tyr levels in our hands. Additionally, a 5′ pre-tRF-Tyr described in response to oxidative stress in the human mammary epithelial cell line, MCF10A, was surprisingly independent of TSEN2 and might also result from self-splicing (46). Altogether, evidence suggests that there are likely other endonucleases or ribozymes, independent of tRNA splicing factors, responsible for the full repertoire of tRFs sequenced under different stress or infection conditions.

We found that *Clp1* knockdown results in decreased MHV68 endpoint titer without impacting ORF50 or gB mRNA expression levels. As true late transcripts like gB require both viral DNA replication and proper expression of the viral late gene transcription complex, the drop in MHV68 titer is likely due to a defect downstream of late gene transcription, perhaps at the stage of translation of late genes, capsid assembly and packaging, or nuclear/cellular egress. We are currently dissecting these possibilities. The role of CLP1 as a proviral factor might suggest that proper regulation of tRNA splicing is necessary for viral replication, although a tRNA-independent activity of CLP1 such as 3′ pre-mRNA processing could be involved (47, 48). The fact that overexpression of *Clp1* was not sufficient to boost MHV68 titers can be interpreted in several ways. It might indicate that a product made downstream of CLP1, such as tRNAs or mRNAs that would theoretically go through multiple other processing steps downstream of CLP1, is the factor important for MHV68 replication. In this scenario, overexpression of the CLP1 RNA kinase might enhance the level of its phosphorylated RNA targets, but the effect could be limited or masked by the limiting expression level or activity of downstream RNA processing factors. In contrast, knockdown of *Clp1* disrupts an essential RNA processing step that cannot be rescued by downstream factors. Careful analysis of changes to tRNA and mRNA processing matched to translational studies will be required to understand the role of Clp1 during MHV68 replication.

Overall, we present a comprehensive profile of tRNAs and tRFs induced by MHV68 and highlight how tRNA processing can be manipulated by and support virus infectivity. Our careful analysis of MHV68-induced tRFs using OTTR-tRAX reveals that these tRFs are derived from both viral tRNAs and host pre-tRNAs, implicating tRNA maturation factors in tRF biogenesis. More work is required to fully understand the functional impact of these infection-induced tRFs. However, in light of the experimental evidence that tRF production is a cellular response to infection (2, 18, 49, 50), is conserved from bacteria to humans (51–53), and can functionally impact gene expression (7, 8, 10, 54, 55), we favor the hypothesis that virus-induced tRFs are not solely byproducts but functional drivers of infection.

## Data Availability

Sequencing data have been deposited in NCBI GEO under identification number GSE255627.

## SUPPLEMENTAL MATERIAL

Supplemental Material mbio.00875-25-s0001.pdf Supplemental figures.

ASM does not own the copyrights to Supplemental Material that may be linked to, or accessed through, an article. The authors have granted ASM a non-exclusive, world-wide license to publish the Supplemental Material files. Please contact the corresponding author directly for reuse.
