# Supplementary material for: Gammaherpesvirus infection triggers the formation of tRNA fragments from premature tRNAs
Source: mBio. 2025 May 30;16(7):e00875-25. doi: 10.1128/mbio.00875-25 (PMC12239594; doi:10.1128/mbio.00875-25)

Supplemental Figure Legends

Supplemental Figure 1. Viability assays on MHV68-infected murine fibroblasts. (A) NIH 3T3 cells were stained with Zombie Violet (Biolegend) and analyzed by FACS and (B) the growth media was assessed for LDH release. (A) For a positive control, we used uninfected NIH 3T3s spiked with heat-killed NIH 3T3 cells (left graph, light blue). Negative controls included both uninfected and infected NIH 3T3 without Zombie Violet (red, pink), as well as uninfected cells with Zombie Violet (gray). Experimental samples (right) include NIH 3T3s mock-infected or infected with MHV68-MR or -R443I at an MOI=5 for 24 hours. (B) Supernatants from NIH 3T3s mock-infected or infected with MHV68-MR or -R443I at an MOI=5 for 24 hours were diluted 1:200 in LDH buffer and analyzed with the LDH-Glo Cytotoxicity Assay (Promega). For a positive control, we used supernatant from NIH 3T3s lysed with 10% Triton-X-100 for 30 min. (C) MC57G fibroblasts were stained with Zombie Violet as in (A). (D) Supernatants from MC57G cultures were analyzed by LDH-Glo as in (B). For a positive control, we used supernatant from MC57Gs lysed with 10% Triton-X-100 for 30 min.

Supplemental Figure 2. 5' tRFs induced by MHV68 infection. Normalized read coverage (5' - 3') from mock (left), MHV68-MR (middle), or MHV68-R443I (right) across tRNA genes. 5' tRFs are defined by tRAX as reads that are within 10 base pairs of the start position, but do not reach the end of the full tRNA sequence. Colors of the coverage defines the specificity of read mapping. Purple "unique" reads uniquely map to the corresponding tRNA transcript sequence. Blue "multitRNA" reads map to the transcript with the corresponding anticodon. Green "multianticodon" reads map only to transcripts of the corresponding tRNA isotype. Red "multiamino" are reads that map to more than one tRNA isotype.

Supplemental Figure 3. 3’ tRFs induced by MHV68 infection. Normalized read coverage (5’ -> 3’) from mock (left), MHV68-MR (middle), or MHV68-R443I (right) across tRNA genes. 3’ tRFs are defined by tRAX as reads that are within 10 base pairs of the end position, but do not reach the start position of the full tRNA sequence. See Supp Fig 1 legend for coloring information.

Supplemental Figure 4. Other tRFs induced by MHV68 infection. Normalized read coverage (5’ -> 3’) from mock (left), MHV68-MR (middle), or MHV68-R443I (right) across tRNA genes. Other tRFs are defined by tRAX as reads that cannot be defined as 5’ or 3’ tRFs. See Supp Fig 1 legend for coloring information.

Supplemental Figure 5. Viral miRNAs and virtRFs. Normalized read coverage across TMERs from the 15-50nt size selected libraries for mock (top), MHV68-MR (middle), and MHV68-R433I (bottom). Color bars below indicate either a viral- tRNA feature (green) or a viral-miRNA feature (orange) and text indicates miRNA nomenclature.

Supplemental Figure 6. Correlative plots comparing pre-tRF expression to parental pre-tRNA transcripts. Log2(FC) values for pre-tRFs from MHV68-MR vs. mock infections are plotted against Log2(FC) values for their parental pre-tRNAs. The r2 value was calculated in Prism using the correlation function.

Supplemental Figure 7. Assessment of Clp1 mRNA levels and siRNA-treated cell viability. (A) RT- qPCR was performed using total RNA from MHV68-MR or MHV68-R443I-infected NIH 3T3s to detect mouse *Clp1* and host *18S* transcripts. Data depicts means +/- SD from three independent experiments, with p-values calculated using raw ∆Ct values and unpaired t-test. (B) NIH 3T3s were treated with non-targeting control siRNAs (siNT), *Clp1*-targeting (siClp1), or *Tsen2*-targeting (siTsen2) siRNAs for 24 h, then mock-infected or infected with MHV68-MR at an MOI=5 for 24 hours. Cells were stained with Zombie Violet (Biolegend) and analyzed by FACS. Our positive control is uninfected NIH 3T3s spiked with heat-killed NIH 3T3 cells (left graph, light blue). (C) Supernatants from the same conditions in (B) were diluted 1:200 in LDH buffer and analyzed with the LDH-Glo Cytotoxicity Assay (Promega). For a positive control, we used supernatant from NIH 3T3s lysed with 10% Triton-X-100 for 30 min.

Supp Figure 1

A

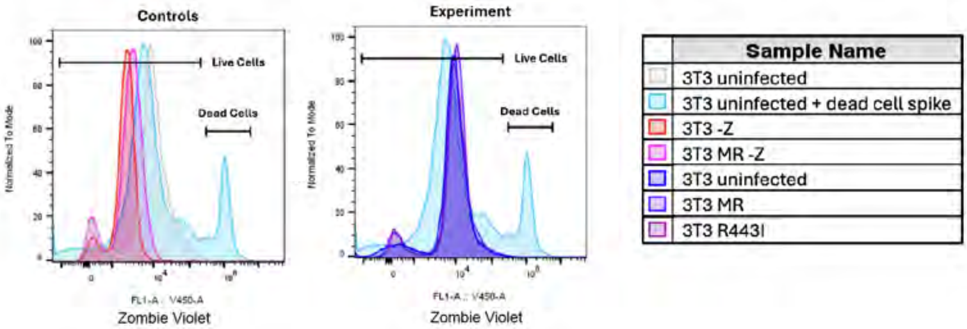

B

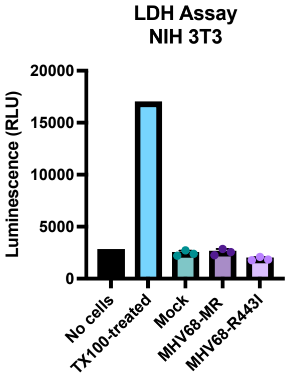

C

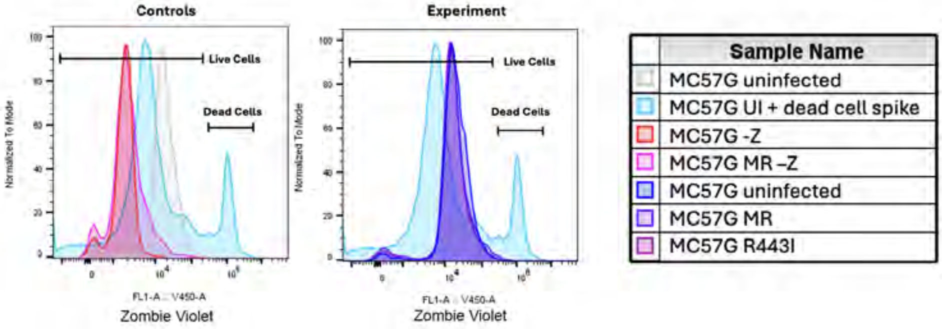

D

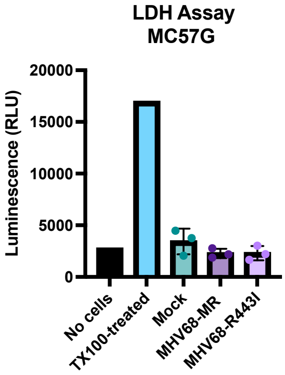

Supp Fig 2

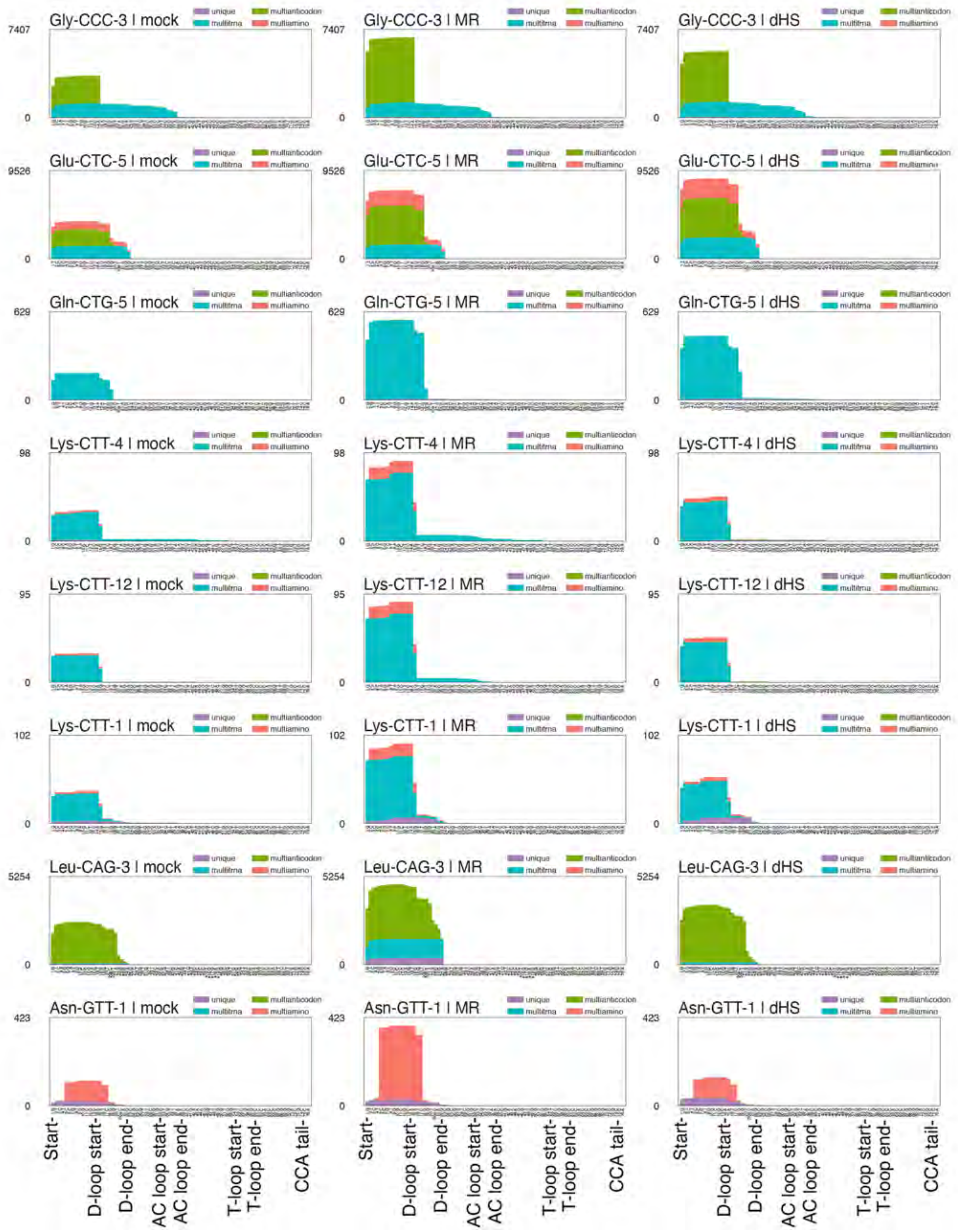

Gly-CCC-3 I mock
Gly-CCC-3 I MR
Gly-CCC-3 I dHS
Glu-CTC-5 I mock
Glu-CTC-5 I MR
Glu-CTC-5 I dHS
Gln-CTG-5 I mock
Gln-CTG-5 I MR
Gln-CTG-5 I dHS
Lys-CTT-4 I mock
Lys-CTT-4 I MR
Lys-CTT-4 I dHS
Lys-CTT-12 I mock
Lys-CTT-12 I MR
Lys-CTT-12 I dHS
Lys-CTT-1 I mock
Lys-CTT-1 I MR
Lys-CTT-1 I dHS
Leu-CAG-3 I mock
Leu-CAG-3 I MR
Leu-CAG-3 I dHS
Asn-GTT-1 I mock
Asn-GTT-1 I MR
Asn-GTT-1 I dHS
unique
multianticodon
multitrna
multiamino
7407
9526
629
98
95
102
5254
423
0
Start-
D-loop start-
D-loop end-
AC loop start-
AC loop end-
T-loop start-
T-loop end-
CCA tail-

Supp Fig 3

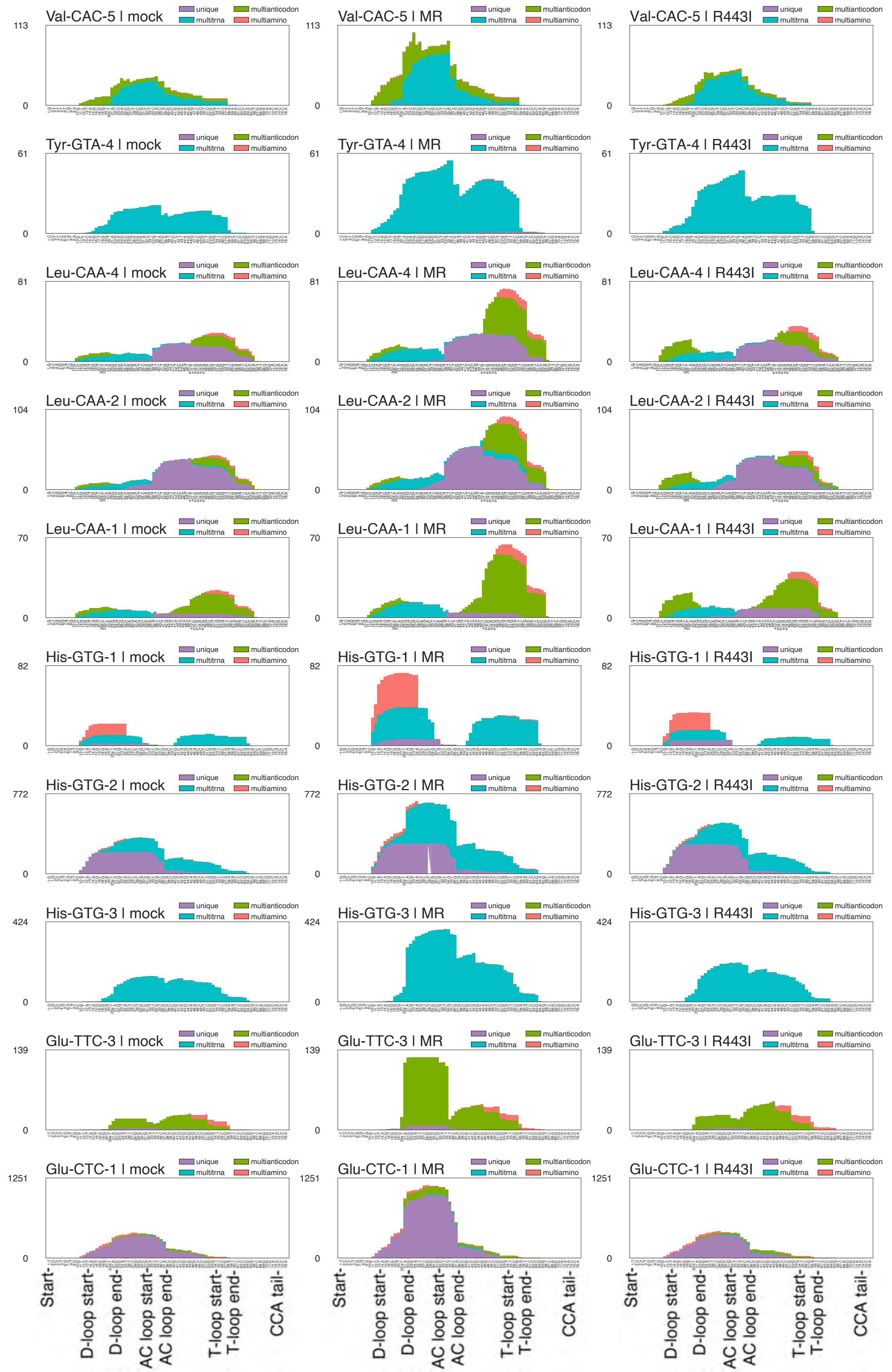

Val-CAC-5 | mock
Val-CAC-5 | MR
Val-CAC-5 | R443I
113
Tyr-GTA-4 | mock
Tyr-GTA-4 | MR
Tyr-GTA-4 | R443I
61
Leu-CAA-4 | mock
Leu-CAA-4 | MR
Leu-CAA-4 | R443I
81
Leu-CAA-2 | mock
Leu-CAA-2 | MR
Leu-CAA-2 | R443I
104
Leu-CAA-1 | mock
Leu-CAA-1 | MR
Leu-CAA-1 | R443I
70
His-GTG-1 | mock
His-GTG-1 | MR
His-GTG-1 | R443I
82
His-GTG-2 | mock
His-GTG-2 | MR
His-GTG-2 | R443I
772
His-GTG-3 | mock
His-GTG-3 | MR
His-GTG-3 | R443I
424
Glu-TTC-3 | mock
Glu-TTC-3 | MR
Glu-TTC-3 | R443I
139
Glu-CTC-1 | mock
Glu-CTC-1 | MR
Glu-CTC-1 | R443I
1251
0
unique
multitrna
multianticodon
multiamino
Start-
D-loop start-
D-loop end-
AC loop start-
AC loop end-
T-loop start-
T-loop end-
CCA tail-

Supp Fig 4

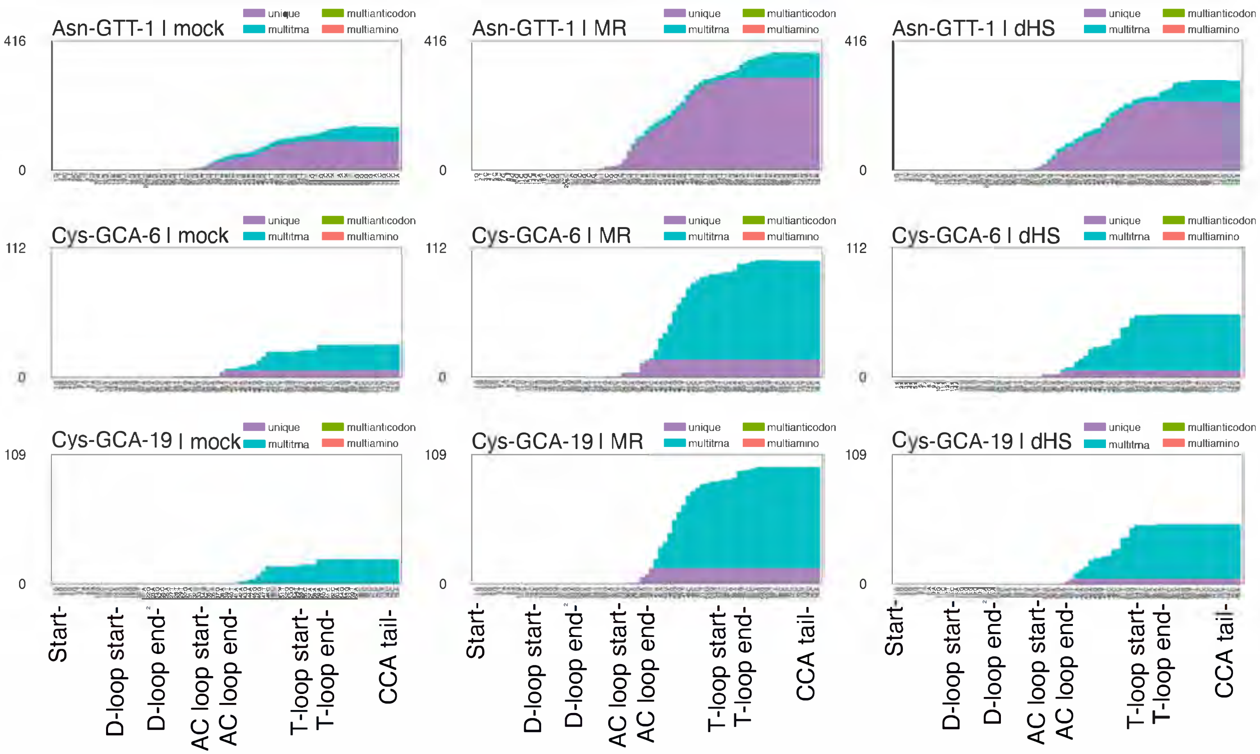
Asn-GTT-1 I mock
Asn-GTT-1 I MR
Asn-GTT-1 I dHS
Cys-GCA-6 I mock
Cys-GCA-6 I MR
Cys-GCA-6 I dHS
Cys-GCA-19 I mock
Cys-GCA-19 I MR
Cys-GCA-19 I dHS
unique
multitrna
multianticodon
multiamino
416
112
109
0
Start-
D-loop start-
D-loop end-
AC loop start-
AC loop end-
T-loop start-
T-loop end-
CCA tail-

Supp Fig 5

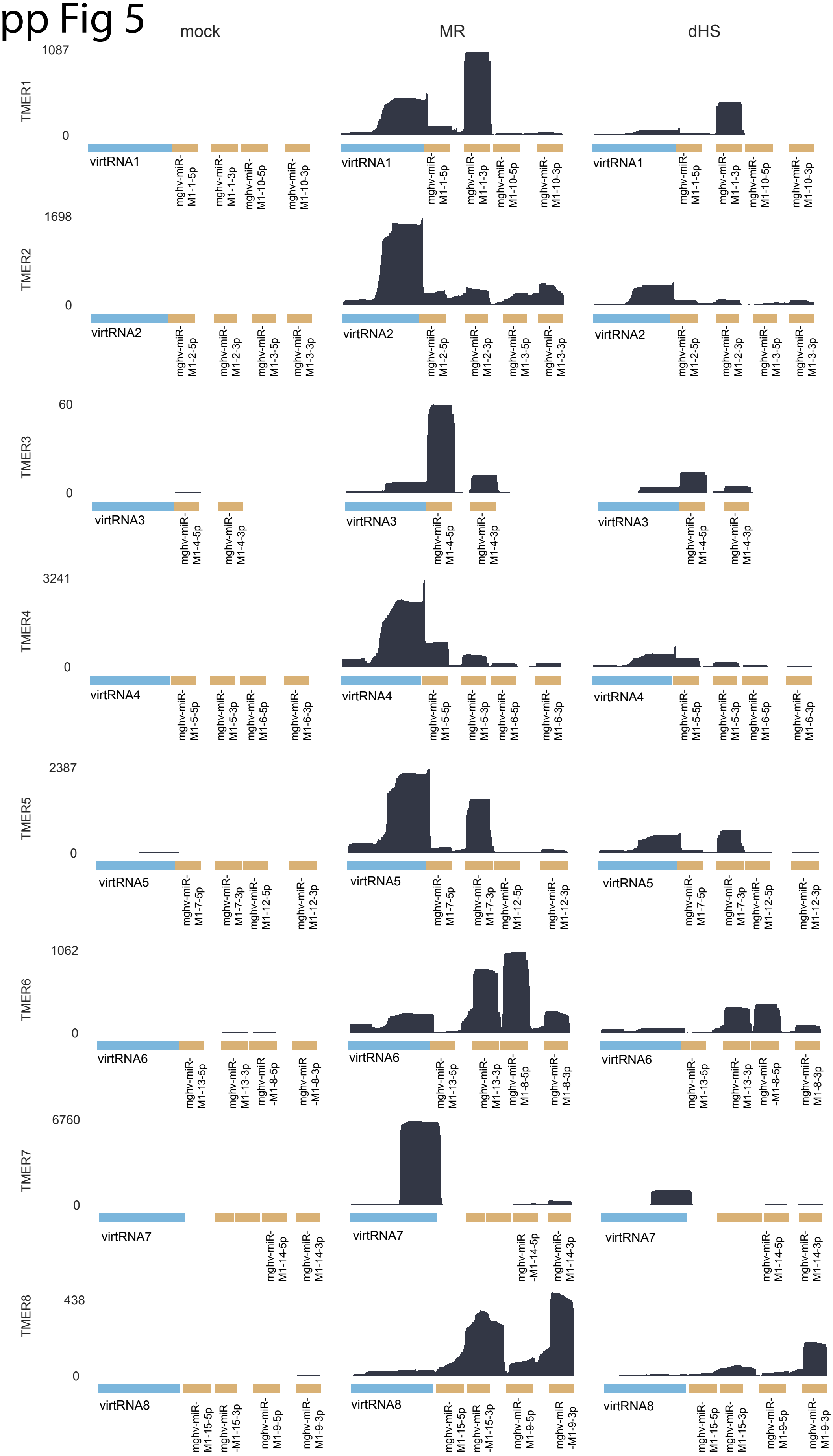
mock
MR
dHS
TMER1
1087
0
virtRNA1
mghv-miR-M1-1-5p
mghv-miR-M1-1-3p
mghv-miR-M1-10-5p
mghv-miR-M1-10-3p
TMER2
1698
0
virtRNA2
mghv-miR-M1-2-5p
mghv-miR-M1-2-3p
mghv-miR-M1-3-5p
mghv-miR-M1-3-3p
TMER3
60
0
virtRNA3
mghv-miR-M1-4-5p
mghv-miR-M1-4-3p
TMER4
3241
0
virtRNA4
mghv-miR-M1-5-5p
mghv-miR-M1-5-3p
mghv-miR-M1-6-5p
mghv-miR-M1-6-3p
TMER5
2387
0
virtRNA5
mghv-miR-M1-7-5p
mghv-miR-M1-7-3p
mghv-miR-M1-12-5p
mghv-miR-M1-12-3p
TMER6
1062
0
virtRNA6
mghv-miR-M1-13-5p
mghv-miR-M1-13-3p
mghv-miR-M1-8-5p
mghv-miR-M1-8-3p
TMER7
6760
0
virtRNA7
mghv-miR-M1-14-5p
mghv-miR-M1-14-3p
TMER8
438
0
virtRNA8
mghv-miR-M1-15-5p
mghv-miR-M1-15-3p
mghv-miR-M1-9-5p
mghv-miR-M1-9-3p

Supp Fig 6

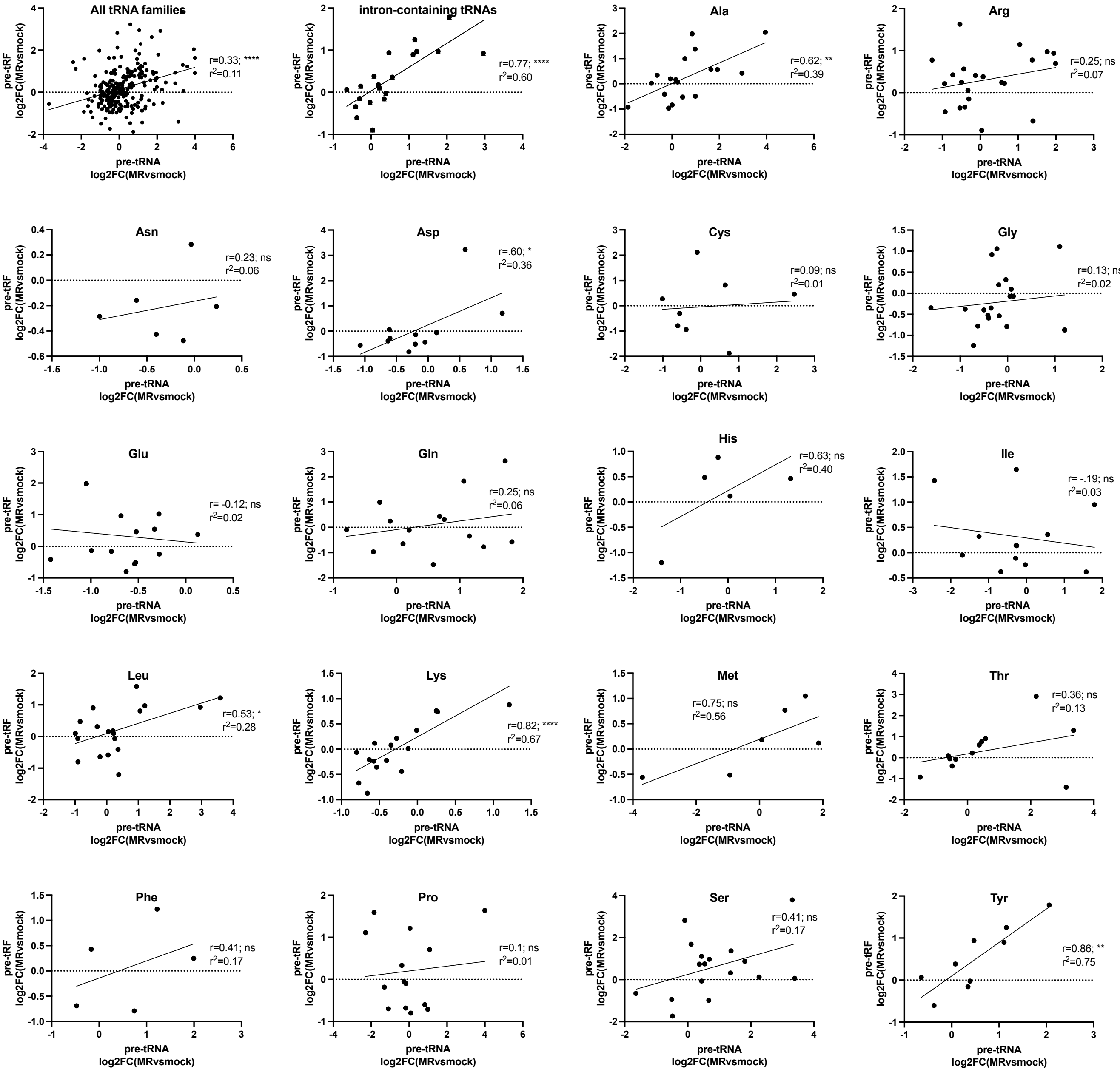
All tRNA families
r=0.33; ****
r^2=0.11
intron-containing tRNAs
r=0.77; ****
r^2=0.60
Ala
r=0.62; **
r^2=0.39
Arg
r=0.25; ns
r^2=0.07
Asn
r=0.23; ns
r^2=0.06
Asp
r=.60; *
r^2=0.36
Cys
r=0.09; ns
r^2=0.01
Gly
r=0.13; ns
r^2=0.02
Glu
r= -0.12; ns
r^2=0.02
Gln
r=0.25; ns
r^2=0.06
His
r=0.63; ns
r^2=0.40
Ile
r= -.19; ns
r^2=0.03
Leu
r=0.53; *
r^2=0.28
Lys
r=0.82; ****
r^2=0.67
Met
r=0.75; ns
r^2=0.56
Thr
r=0.36; ns
r^2=0.13
Phe
r=0.41; ns
r^2=0.17
Pro
r=0.1; ns
r^2=0.01
Ser
r=0.41; ns
r^2=0.17
Tyr
r=0.86; **
r^2=0.75
pre-tRF
log2FC(MRvsmock)
pre-tRNA
log2FC(MRvsmock)

Supp Fig 7

A

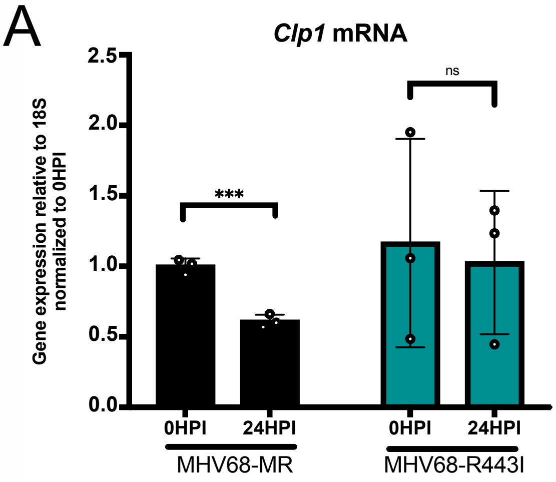

B

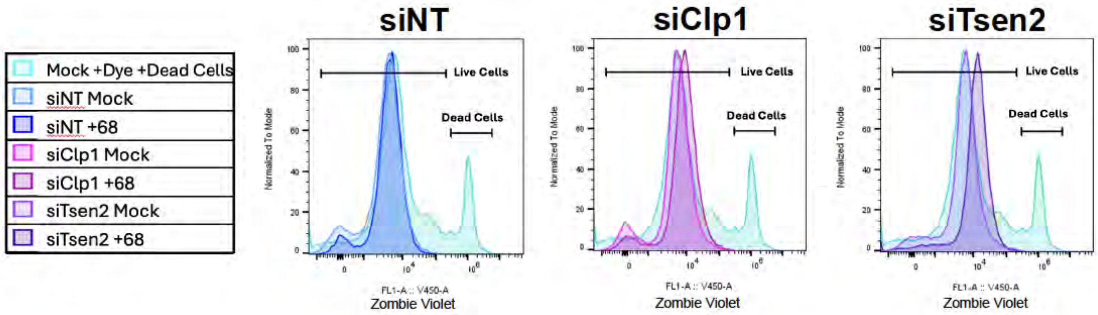

C

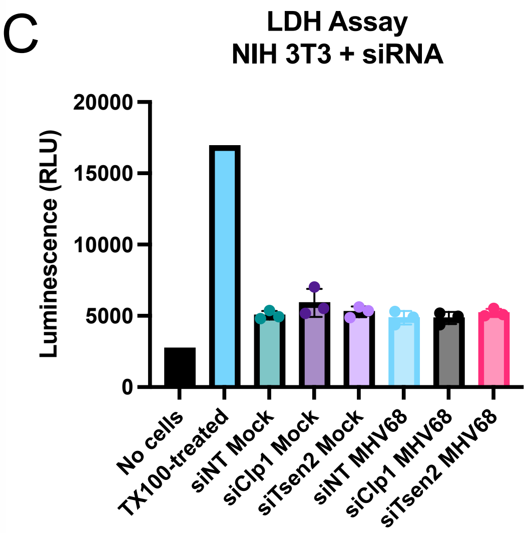

Supplement: Supplemental Material — Supplemental figures. [file mbio.00875-25-s0001.pdf]
